# Oscillating lncRNA *Platr4* regulates *NLRP3* inflammasome to ameliorate nonalcoholic steatohepatitis in mice

**DOI:** 10.7150/thno.50281

**Published:** 2021-01-01

**Authors:** Yanke Lin, Shuai Wang, Lu Gao, Ziyue Zhou, Zemin Yang, Jingpan Lin, Shujing Ren, Huijie Xing, Baojian Wu

**Affiliations:** 1 College of Pharmacy, Jinan University, Guangzhou 510632, China.; 2 Integrated Chinese and Western Medicine Postdoctoral research station, Jinan University, Guangzhou, 510632, China.; 3 Institution of Laboratory Animal, Jinan University, 601 Huangpu Avenue West, Guangzhou, China.

**Keywords:** *Platr4*, * NLRP3* inflammasome, NF-κB, RXR, Steatohepatitis

## Abstract

**Background:** Understanding the molecular events and mechanisms underlying development and progression of nonalcoholic steatohepatitis is essential in an attempt to formulating a specific treatment. Here, we uncover Platr4 as an oscillating and NF-κB driven lncRNA that is critical to the pathological conditions in experimental steatohepatitis

**Methods:** RNA-sequencing of liver samples was used to identify differentially expressed lncRNAs. RNA levels were analyzed by qPCR and FISH assays. Proteins were detected by immunoblotting and ELISA. Luciferase reporter, ChIP-sequencing and ChIP assays were used to investigate transcriptional gene regulation. Protein interactions were evaluated by Co-IP experiments. The protein-RNA interactions were studied using FISH, RNA pull-down and RNA immunoprecipitation analyses

**Results:** Cyclic expression of Platr4 is generated by the core clock component Rev-erbα via two RevRE elements (i.e., -1354/-1345 and -462/-453 bp). NF-κB transcriptionally drives Platr4 through direct binding to two κB sites (i.e., -1066/-1056 and -526/-516 bp), potentially accounting for up-regulation of Platr4 in experimental steatohepatitis. Intriguingly, *Platr4* serves as a circadian repressor of *Nlrp3* inflammasome pathway by inhibiting NF-κB-dependent transcription of the inflammasome components Nlrp3 and Asc. Loss of Platr4 down-regulates *Nlrp3* inflammasome activity in the liver, blunts its diurnal rhythm, and sensitizes mice to experimental steatohepatitis, whereas overexpression of *Platr4* ameliorates the pathological conditions in an *Nlrp3*-dependent manner. Mechanistically, *Platr4* prevents binding of the NF-κB/Rxrα complex to the κB sites via a physical interaction, thereby inhibiting the transactivation of Nlrp3 and Asc by NF-κB.

**Conclusions:** *Platr4* functions to inactivate *Nlrp3* inflammasome via intercepting NF-κB signaling. This lncRNA might be an attractive target that can be modulated to ameliorate the pathological conditions of steatohepatitis.

## Introduction

Nonalcoholic fatty liver disease (NAFLD) is the most common chronic liver disorder, with a high incidence in developed countries (20-30% prevalence) and an increasing incidence in developing countries 1. Nonalcoholic steatohepatitis (NASH) is a severe form of NAFLD, characterized by fat accumulation in the liver and varying degrees of inflammation and fibrosis 2. NASH may progress to liver cirrhosis and hepatocellular carcinoma, and affected patients tend to have an increased liver-related mortality 3. Notably, NASH is becoming a leading indication for liver transplantation in the United States 4,5. The pathogenesis of NASH is poorly understood and there are no specific drugs for disease treatment, although some risk factors (e.g., oxidative stress and metabolic morbidities such as dyslipidemia, obesity, insulin resistance and type 2 diabetes) have been defined 4. Accordingly, the goal of current interventions for NAFLD and NASH is to mitigate the associated metabolic comorbidities 4. Nevertheless, “two-hit” and “multiple-hit” hypotheses have been proposed to account for the pathogenesis of NASH 6,7. In both theories, aberrant inflammation is implicated the development and progression of NASH. Therefore, studies to clarify the specific mechanisms for deregulated inflammation are critical for development of new therapeutic strategies to prevent and manage steatohepatitis.

The innate immunity recognizes not only the pathogen products (pathogen-associated molecular patterns, PAMPs) but also endogenous danger signals (damage-associated molecular patterns, DAMPs), and provides immediate defense by mounting strong inflammatory responses 8. NLRP3 (NOD-, LRR- and pyrin domain-containing protein 3) inflammasome plays a central role in these responses. It is a multi-protein complex consisting of NLRP3 (a sensor), ASC (apoptosis-associated speck-like protein containing a CARD, an adaptor), and pro-caspase-1 (an effector). NLRP3 inflammasome activation is a two-step process, namely, it must be first primed and then can be activated 9. The priming step, triggered by the signal 1 (e.g., PAMPs and cytokines), mainly serves to up-regulate the expression of the inflammasome components via NF-κB activation and gene transcription 9. Signal 2 (e.g., PAMPs and DAMPs such as particulates, crystals and adenosine triphosphate (ATP)) triggers various upstream signaling events including potassium efflux, calcium flux, mitochondrial dysfunction and lysosomal disruption which induce the complex formation of and activation of NLRP3 inflammasome 9. The inflammasome complex promotes the cleavage of pro-caspase-1 (to form caspase-1) and ensuing maturation of the proinflammatory cytokines IL-1β and IL-18. Although NLRP3 inflammasome is essential for immune defense against microbial infection and cellular damage, excessive and deregulated activation can lead to the development of pathological inflammation that may be a cause of a wide range of diseases, including gout, atherosclerosis, diabetes, rheumatoid arthritis and Alzheimer's disease 10. Additionally, recent years of studies have revealed a tight association of NASH pathogenesis with aberrant activation of NLRP3 inflammasome 11. Inhibition of NLRP3 inflammasome activity is shown to reduce liver inflammation and fibrosis in experimental NASH 12. Thus, there has been a great interest to understand how the NLRP3 inflammasome is regulated in NASH.

Most facets of physiology and behaviors in mammals are governed by circadian rhythms (with a period of about 24-h). The immune functions are no exception as manifested by circadian rhythmicity in immune cell counts and cytokine levels as well as diurnal variations in the response to endotoxin and the circadian control of allergic reactions 13. Notably, *NLRP3* transcription and expression are oscillating, accounting for circadian rhythmicity in *NLRP3* inflammasome pathway 14. Circadian rhythms are driven and maintained by the circadian clock system with a hierarchical organization. The central clock (pacemaker) is located at the suprachiasmatic nucleus of the hypothalamus and peripheral clocks are present in the peripheral tissues. The central clock synchronizes the peripheral clocks via nervous and hormonal pathways 15. On the other hand, local clocks in peripheral tissues such as liver and kidney can be entrained by external stimuli (e.g., food) independent of the central clock 16. Strikingly, approximately 43% of all protein coding genes are clock-controlled genes 17. Molecular components of circadian clock maintain their oscillations using a negative feedback mechanism (so-called “transcriptional-translational feedback loop”) 18. In the main feedback loop, the positive components [BMAL1 (brain and muscle ARNT-like 1) and CLOCK (circadian locomotor output cycles kaput)] form a heterodimer to activate gene transcription of negative components [period (PER) 1,2 and cryptochrome (CRY) 1,2] via direct binding to the E-box *cis-*regulatory element (CACGTG) 19. Following protein accumulation to a high level, PERs and CRYs in turn inhibit the activity of the positive components, thereby down-regulating their own expression levels. Once the proteins of negative components are degraded, their inhibitory actions no longer exist. Positive components can bind to target genes to start a new cycle of transcription and translation 18. The nuclear heme receptor REV-REBα is another clock component that regulates circadian rhythms via inhibiting the transcription and expression of BMAL1 18.

Retinoid X receptors [including RXRα (NR2B1), RXRβ (NR2B2) and RXRγ (NR2B3)] are essential members of the nuclear receptor (NR) superfamily. RXRs function as transcription factors and regulate gene transcription by forming homodimers or heterodimers with other NRs. Like many other NRs, RXRs are ligand-responsive and can be activated by endogenous 9-*cis-*retinoic acid and fatty acids as well as synthetic retinoids 20. Of three isotypes, RXRα perhaps plays a more important role in biology and physiology because disruption of RXRα leads to embryonic lethality, while deficiency of RXRβ or RXRγ is less severe 20. RXRs display a tissue-specific difference in expression. Notably, RXRα is predominantly expressed in the liver, intestine and kidney, as well as in myeloid cells 21. RXRs have been implicated in regulation of a variety of biological processes and are attractive drug targets for cancer and metabolic diseases 22. Intriguingly, NF-κB may exist constitutively with RXR (an NF-κB active complex); however, this complex becomes inactive in the presence of an RXR ligand 23.

A dominant portion of mammalian genome is transcribed as ncRNAs (non-coding RNAs). LncRNAs (long non-coding RNAs) are a class of ncRNAs that have over 200 nucleotides. They have emerged as major regulators of the transcriptional process by interacting physically with DNA, other RNA or proteins 24. Many lncRNAs have been implicated in the development of liver diseases and in regulation of inflammation and immunity 25-28. For instance, *MALAT1* lncRNA may contribute to development of fibrosis in NAFLD through a mechanism involving the chemokine CXCL5 29. In addition, lncRNAs have potential impact on circadian biology, and in turn they may be under the control of circadian clock 30,31. It is thus hypothesized that circadian *NLRP3* inflammasome activity and steatohepatitis (an *NLRP3*-related disease) may be regulated by an oscillating lncRNA. Platr (pluripotency-associated transcript) family consists of 32 members (i.e., Platr1 to -32), which were identified in an RNA-seq screen of embryonic stem cells (ESCs) and implicated in maintenance of the ESC gene expression profile 32. In MMTV-Neu-NDL breast cancer model, *Platr4* was upregulated more than 4-fold and may be associated with tumor progression 33. In this study, we uncover *Platr4* as an oscillating lncRNA deregulated in mice with steatohepatitis. Oscillation of *Platr4* is driven by the circadian clock component Rev-erbα. We found that *Platr4* serves as a circadian repressor of *Nlrp3* inflammasome activity by inhibiting transcription and expression of the inflammasome components *Nlrp3* and Asc. Loss of *Platr4* sensitizes mice to experimental steatohepatitis, whereas overexpression of *Platr4* ameliorates the pathological conditions. Mechanistically, *Platr4* prevents binding of the NF-κB/Rxrα complex to the κB sites via a physical interaction, thereby inhibiting the transactivation of *Nlrp3* and *Asc* by NF-κB. We thus propose that *Platr4* might be an attractive target for management of steatohepatitis.

## Results

### Identification of *Platr4* as an oscillating and steatohepatitis-related lncRNA

**Figure 1:**
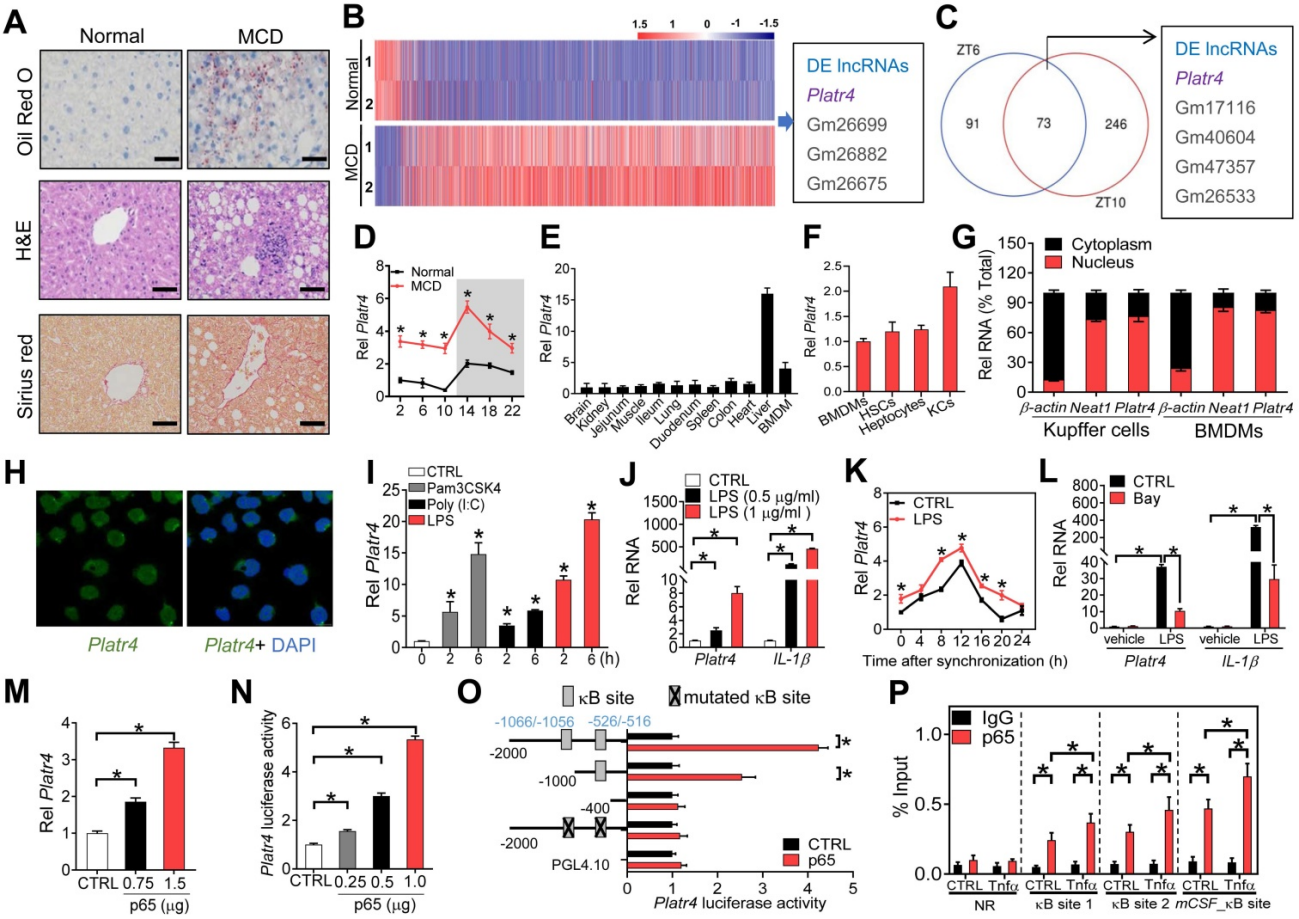
***Platr4* is an oscillating and steatohepatitis-related lncRNA whose expression is driven by NF-κB. (A)** Representative micrographs of Oil Red O, H&E and Sirius red staining of liver sections from normal mice and mice with MCD-induced steatohepatitis. Scale bar = 50 μm. **(B)** Heatmap diagram for differentially expressed (DE) genes in livers from normal mice and mice with MCD-induced steatohepatitis. **(C)** Venn diagram showing the numbers of differentially expressed lncRNAs (DE lncRNAs) in the livers of wild-type and *Rev-erbα^-/-^* mice at ZT6 and ZT10. Data are available upon request.** (D)** Diurnal hepatic *Platr4* expression in normal mice and mice with MCD-induced steatohepatitis. Data are mean ± SD (*n* = 8). *p <0.05 versus normal mice at individual time points (two-way ANOVA and Bonferroni post hoc test).** (E)** Expression comparisons of *Platr4* in the various tissues and BMDMs of wild-type mice. Data are mean ± SD (*n* = 3).** (F)** Expression comparisons of *Platr4* in BMDMs, hepatic stellate cells (HSCs), hepatocytes and Kupffer cells (KCs) derived from wild-type mice. Data are mean ± SD (*n* = 3). **(G)** Relative cytoplasm and nuclear expression of *Platr4* in Kupffer cells and BMDMs of wild-type mice. Data are mean ± SD (*n* = 3).** (H)** FISH analysis of subcellular *Platr4* (green) location in BMDMs from wild-type mice.** (I)** Effects of Pam3CSK4 (a TLR1/2 agonist, 100 nM), Poly (I:C) (a TLR3 agonist, 25 µg/ml) and LPS (a TLR4 agonist, 500 ng/ml) on *Platr4* expression in BMDMs. Data are mean ± SD (*n* = 3). *p <0.05 versus CTRL (one-way ANOVA and Bonferroni post hoc test).** (J)** Dose-dependent effects of LPS (0.5 and 1 μg/ml) on *Platr4* and *IL-1β* mRNA in BMDMs. Data are mean ± SD (*n* = 3). *p <0.05 (one-way ANOVA and Bonferroni post hoc test).** (K)** Effects of LPS (500 ng/ml) on *Platr4* expression in synchronized BMDMs. Data are mean ± SD (*n* = 3). *p <0.05 versus control at individual time points (two-way ANOVA and Bonferroni post hoc test).** (L)** Effects of Bay 11-7082 on *Platr4* and *IL-1β* mRNA in unstimulated or LPS-stimulated BMDMs. Data are mean ± SD (*n* = 3). *p <0.05 (two-way ANOVA and Bonferroni post hoc test).** (M)** Dose-dependent effects of p65 plasmid (0.75 or 1 μg) on *Platr4* expression in BMDMs. Data are mean ± SD (*n* = 3). *p <0.05 (one-way ANOVA and Bonferroni post hoc test).** (N)** Dose-dependent effects of p65 plasmid (0.25, 0.5 or 1 μg) on 2.1 kb *Platr4* reporter activity in BMDMs. Data are mean ± SD (*n* = 6). *p <0.05 (one-way ANOVA and Bonferroni post hoc test).** (O)** Effects of p65 on the activities of different versions of *Platr4-Luc* reporters. Data are mean ± SD (*n* = 6). *p <0.05 (t-test).** (P)** Recruitment of p65 protein to the κB sites (-1066/-1056 bp and -526/-516 bp) of *Platr4* promoter in unstimulated or Tnfα-stimulated BMDMs. Data are mean ± SD (*n* = 3). *p <0.05 (two-way ANOVA and Bonferroni post hoc test). NR, non-binding region.

Mice were fed a methionine-choline-deficient diet (MCD) to induce steatohepatitis. Disease model was confirmed by histopathological examinations (Figure 1A) and biochemical analyses (Figures S1-2). According to transcriptome analysis, *Platr4* (Gencode ID: ENSMUSG00000097639.1) was up-regulated (a > 5-fold increase) in mice with MCD-induced steatohepatitis (Figure 1B). As expected, MCD-associated differentially expressed genes converged on inflammation- and nutrition metabolism-related pathways (Figure S3). *Platr4* was also deregulated in the livers of *Rev-erbα^-/-^* mice (Figure 1C). *Rev-erbα^-/-^* mice were used to identify oscillating (or circadian clock-controlled) lncRNAs because Rev-erbα is a core clock component. Quantitative polymerase chain reaction (qPCR) assays confirmed that *Platr4* was rhythmically expressed in the liver with a peak level at ZT14, and indeed up-regulated in MCD mice (Figure 1D). Coding potential analyses indicated *Platr4* as a “real” noncoding transcript (i.e., does not code a protein) (Figure S4). Notably, *Platr4* was predominantly expressed in the liver among various major tissues (Figure 1E). All three liver cells (i.e., Kupffer cells, hepatocytes and hepatic stellate cells) expressed *Platr4* (Figure 1F). For Kupffer cells, the copy number of *Platr4* was determined to be 40 (i.e., 40 copies per cell). Moderate expression of *Platr4* was also seen in bone marrow-derived macrophages (BMDMs) (Figure 1E). In addition, like *Neat1* (a known nuclear lncRNA), *Platr4* was primarily localized in the nuclei of Kupffer cells and BMDMs (Figure 1G-H). Altogether, the oscillating lncRNA *Platr4* is liver-specific and may have a regulatory role in development of steatohepatitis.

### NF-κB activation promotes *Platr4* expression

NF-κB activation is implicated in development of steatohepatitis 34. We wondered whether up-regulation of *Platr4* is associated with NF-κB activation in experimental steatohepatitis. Expression of *Platr4* in BMDMs was induced by Pam3CSK4 (an activator of NF-κB mediated by TLR1/2 receptors), Poly (I:C) (an activator of NF-κB mediated by TLR3) and lipopolysaccharide (LPS, an activator of NF-κB mediated by TLR4) in a time-dependent manner (Figure 1I). Also, induction of expression of* Platr4* and* IL-1β* (a known NF-κB target gene) by LPS was dose-dependent (Figure 1J). Similar inductive effects of LPS on *Platr4* were observed in synchronized BMDMs (Figure 1K). In contrast, Bay 11-7082 (a specific NF-κB inhibitor) inhibited LPS-stimulated expression of *Platr4* and *IL-1β* (Figure 1L). These findings suggested a positive role of NF-κB in controlling *Platr4* expression. Supporting this, overexpression of p65 (an NF-κB subunit) enhanced the expression of *Platr4* in BMDMs (Figure 1M). In line with a positive regulation effect, p65 dose-dependently induced the promoter activity of a 2.1 kb *Platr4-Luc* reporter (Figure 1N). We further searched for κB sites to which NF-κB binds and thereafter activates target gene transcription. Promoter sequence analysis identified two κB sites (i.e., -1066/-1056 bp and -526/-516 bp) in *Platr4* promoter. Truncation and mutation experiments indicated that the two κB sites were essential for NF-κB binding and activity (Figure 1O). Recruitment of p65 to these two κB sites was confirmed by ChIP (chromatin immunoprecipitation) assays (Figure 1P). Of note, p65 recruitment was enhanced by recombinant TNFα protein (Figure 1P). Altogether, NF-κB drives expression of *Platr4* via a transcriptional activation mechanism, and up-regulation of *Platr4* in experimental steatohepatitis may be driven by activated NF-κB.

### Clock component Rev-erbα regulates rhythmic expression of *Platr4* in normal mice

**Figure 2:**
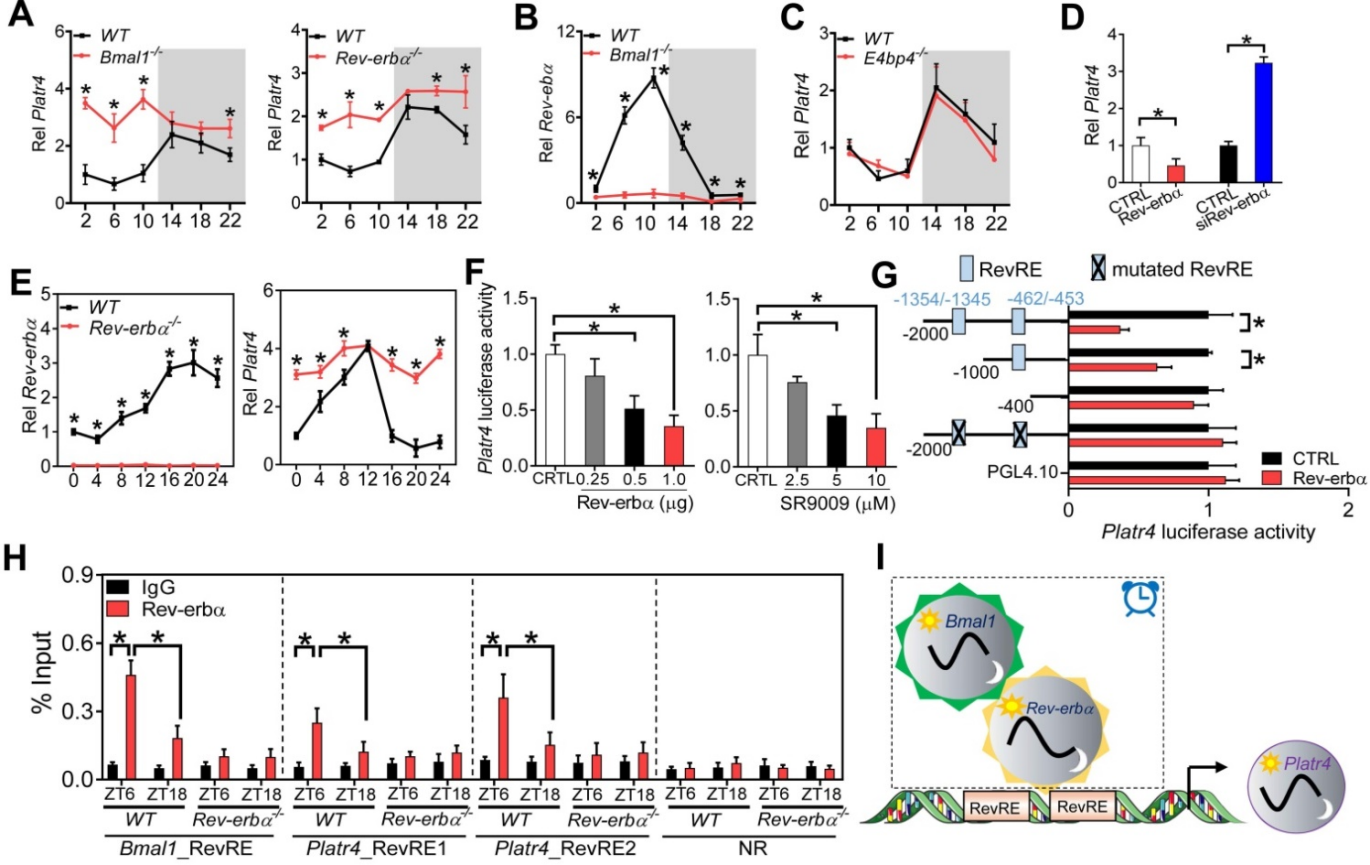
** Rev-erbα regulates rhythmic expression of *Platr4* in normal mice. (A)** Hepatic *Platr4* expression in the *Rev-erbα^-/-^*, *Bmal1^-/-^* and wild-type (WT) mice at 6 circadian time points. Data are mean ± SD (*n* = 5). *p <0.05 versus WT at individual time points (two-way ANOVA and Bonferroni post hoc test).** (B)** Hepatic *Rev-erbα* expression in the *Bmal1^-/-^* and WT mice at 6 circadian time points. Data are mean ± SD (*n* = 5). *p <0.05 versus WT at individual time points (two-way ANOVA and Bonferroni post hoc test).** (C)** *Platr4* expression in the livers of *E4bp4^-/-^* and WT mice at 6 circadian time points. Data are mean ± SD (*n* = 5).** (D)** Effects of Rev-erbα overexpression and knockdown on *Platr4* expression in BMDMs. Data are mean ± SD (*n* = 3). *p <0.05 (t-test).** (E)** Temporal expression of *Rev-erbα* (left panel) and *Platr4* (right panel) in synchronized BMDMs derived from *Rev-erbα^-/-^* and WT mice. Data are mean ± SD (*n* = 3). *p <0.05 versus WT at individual time points (two-way ANOVA and Bonferroni post hoc test).** (F)** Effects of Rev-erbα overexpression (left panel) and SR9009 (a Rev-erbα agonist, right panel) on the 2.1-kb *Platr4* promoter activity in BMDMs. Data are mean ± SD (*n* = 6). *p <0.05 (one-way ANOVA and Bonferroni post hoc test).** (G)** Effects of Rev-erbα on the activities of different versions of *Platr4-Luc* reporters. Data are mean ± SD (*n* = 6). *p <0.05 (t-test).** (H)** Recruitment of Rev-erbα protein to the two RevRE sites (-1354/-1345 and -462/-453 bp) of *Platr4* promoter in livers derived from WT and *Rev-erbα^-/-^* mice at ZT6 and ZT18. Data are mean ± SD (*n* = 3). *p <0.05 (two-way ANOVA and Bonferroni post hoc test).** (I)** Schematic diagram showing the potential mechanism for rhythmic expression of *Platr4*. NR, non-binding region.

Circadian expression of most genes is driven by molecular components of circadian clock via transcriptional actions on their *cis-*elements (E-box, D-box and RevRE or RORE) 35,36. We thus investigated the roles of these three *cis-*elements in rhythmic expression of *Platr4* using *Bmal1^-/-^*, *E4bp4^-/-^* and *Rev-erbα^-/-^* mice. Bmal1, E4bp4 and Rev-erbα are representative clock components that bind to E-box, D-box and RevRE, respectively. Bmal1 functions as a transcriptional activator, while both E4bp4 and Rev-erbα (also known to be activated by Bmal1) are repressors 36. Hepatic* Platr4* was up-regulated and its rhythm was blunted (with a decrease in amplitude) in *Bmal1^-/-^* and in *Rev-erbα^-/-^* mice (Figure 2A). As expected, *Rev-erbα* (as a Bmal1 target gene) was markedly suppressed and its rhythm was lost in *Bmal1^-/-^* mice (Figure 2B). In contrast, hepatic* Platr4* was unaffected in *E4bp4^-/-^* mice (Figure 2C). These data suggested that Rev-erbα directly represses circadian expression of *Platr4*, and that the repressive effect of Bmal1 on *Platr4* is attained indirectly via its target Rev-erbα. Supporting a negative regulation mechanism, Rev-erbα overexpression in BMDMs led to reduced *Platr4* expression, while Rev-erbα knockdown promoted the expression of* Platr4* (Figure 2D). Consistently, Rev-erbα ablation up-regulated *Platr4*, and dampened its rhythm in synchronized BMDMs (Figure 2E). Furthermore, Rev-erbα dose-dependently inhibited the 2.1-kb *Platr4* promoter activity (Figure 2F). Similar dose-dependent inhibitory effects were observed for SR9009 (a Rev-erbα agonist) (Figure 2F). Two RevRE elements (located at -1354/-1345 bp and -462/-453 bp) in *Platr4* promoter were identified to be critical for Rev-erbα action based on promoter analysis, truncation and mutation experiments (Figure 2G). ChIP assays confirmed that hepatic Rev-erbα protein was recruited to the RevRE elements of *Platr4* in wild-type mice in a circadian time-dependent manner (Figure 2H). Notably, Rev-erbα recruitment was more extensive at ZT6 than at ZT18 (Figure 2H). However, Rev-erbα recruitment was reduced and its time-dependency was abolished in *Rev-erbα^-/-^* mice (Figure 2H). Altogether, Rev-erbα periodically trans-represses *Platr4*, contributing to its diurnal rhythmicity in normal mice (Figure 2I).

### *Platr4* inhibits *Nlrp3* inflammasome activation in macrophages

**Figure 3:**
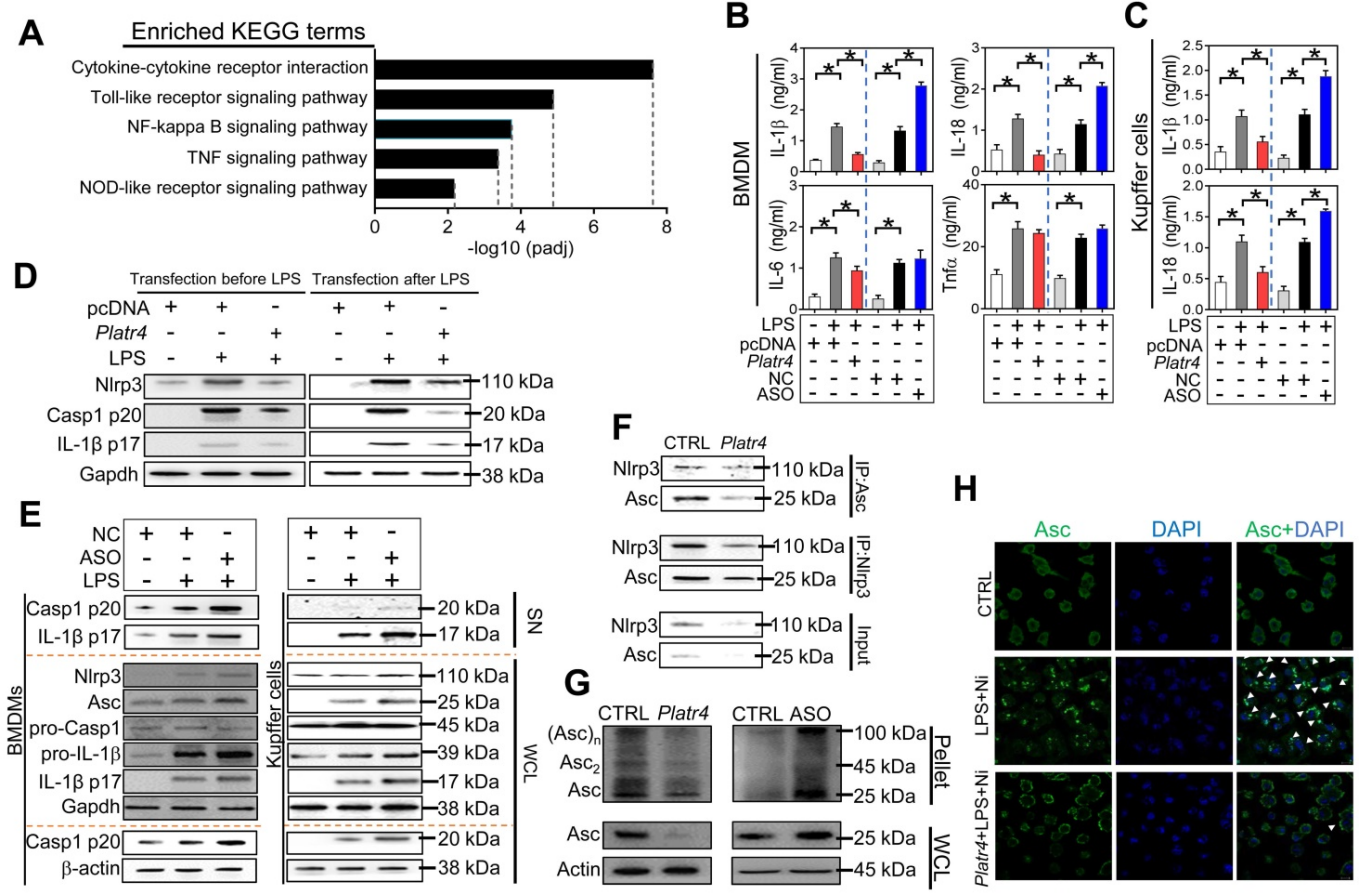
***Platr4* inhibits *Nlrp3* inflammasome activation in macrophages. (A)** KEGG analysis of *Platr4*-induced differentially expressed genes in LPS-primed BMDMs. **(B)** Effects of *Platr4* overexpression and knockdown on production of inflammatory cytokines (IL-1β, IL-18, IL-6 and Tnfα) in LPS-stimulated BMDMs. Data are mean ± SD (*n* = 3). *p <0.05 (one-way ANOVA and Bonferroni post hoc test).** (C)** Effects of *Platr4* overexpression and knockdown on production of IL-1β and IL-18 in LPS-stimulated Kupffer cells. Data are mean ± SD (*n* = 3). *p <0.05 (one-way ANOVA and Bonferroni post hoc test).** (D)** Effects of *Platr4* on protein levels of* Nlrp3*, active caspase 1 (p20) and mature IL-1β (p17) in LPS/nigericin-stimulated BMDMs. LPS was added before or after *Platr4* transfection for 8 h, followed by nigericin addition for 30 min (added last).** (E)** Effects of *Platr4* knockdown (by ASO) on *Nlrp3* inflammasome-related proteins in LPS/nigericin-stimulated BMDMs (left panel) and Kupffer cells (right panel). **(F)** Effects of* Platr4* overexpression on interactions between *Nlrp3* and *Asc* proteins in LPS/nigericin-stimulated BMDMs. **(G)** Effects of *Platr4* overexpression and knockdown on *Asc* oligomerization in LPS/nigericin-stimulated BMDMs. **(H)** Effects of *Platr4* on formation of *Asc* specks in LPS/nigericin-stimulated BMDMs. The concentrations of LPS and nigericin for macrophage treatment were 500 ng/ml and 10 μM, respectively. SN, supernatant. WCL, whole cell lysate. NC, negative control. Ni, nigericin.

To explore the immune function of *Platr4*, *Platr4*-overexpressed and control BMDMs were subjected RNA-seq and KEGG pathway analyses. Differentially expressed genes were enriched in NF-κB activation-related pathways (i.e., NF-κB, Toll-like receptor, TNF, NOD-like receptor signaling pathways, and cytokine-cytokine receptor interaction), suggesting potential involvement of *Platr4* in regulation of NF-κB activation and related immune responses (Figures 3A-S5). Overexpression of *Platr4* decreased, whereas knockdown of *Platr4* (by an antisense oligonucleotide, ASO) increased, the protein levels of the inflammatory cytokines IL-1β and IL-18 in LPS-stimulated BMDMs (Figure 3B). By contrast, *Platr4* had a milder effect on IL-6 or Tnfα (Figure 3B). Negative regulation of both IL-1β and IL-18 by *Platr4* was confirmed in LPS-stimulated Kupffer cells (hepatic macrophages) (Figure 3C). IL-1β and IL-18 production are mainly dependent on caspase-1 activity which is under the control of *NLRP3* inflammasome 37,38. We thus tested whether *Platr4* can regulate *NLRP3* inflammasome activation. Activation of *NLRP3* inflammasome involves two critical processes, namely, a priming step to increase *NLRP3* expression (via NF-κB activation) and an assembling step to form the inflammasome assembly 39. *Platr4* overexpression prior to LPS priming inhibited the generation of active caspase-1 (p20) and mature IL-1β (p17) in BMDMs (Figure 3D). Similar inhibitory effects of *Platr4* on production of active caspase-1 and mature IL-1β were also observed in LPS-primed BMDMs (Figure 3D). This suggested potential actions of *Platr4* on both priming and assembling stages of inflammasome activation. We went on to examine the effects of *Platr4* on three components of *Nlrp3* inflammasome (i.e., *Nlrp3*, *Asc* and pro-caspase-1). *Platr4* knockdown did not affect the expression of pro-caspase-1, but induced the proteins of *Nlrp3*, pro-IL-1β and *Asc* accompanied by elevated cleaved caspase 1 and mature IL-1β in LPS-stimulated BMDMs and Kupffer cells (Figure 3E). After priming, *NLRP3* and *ASC* are assembled via oligomerization to form a large protein aggregate (termed “speck”), a hallmark of inflammasome assembly and activation 40. Overexpression of *Platr4* led to a reduced protein interaction between *Nlrp3* and *Asc* in BMDMs (Figure 3F). Further, *Platr4* suppressed the formation of *Asc* oligomer, and *Platr4* deficiency promoted *Asc* oligomerization (Figure 3G). Moreover, overexpression of *Platr4* resulted in a marked reduction in the number of *Asc* specks upon LPS/nigericin stimulation (Figure 3H). Taken together, *Platr4* functions as a negative regulator of *Nlrp3* inflammasome activation probably through repressing the expression of *Nlrp3* and Asc.

### Loss of *Platr4* blunts the oscillation of *Nlrp3* inflammasome and sensitizes mice to experimental steatohepatitis

**Figure 4:**
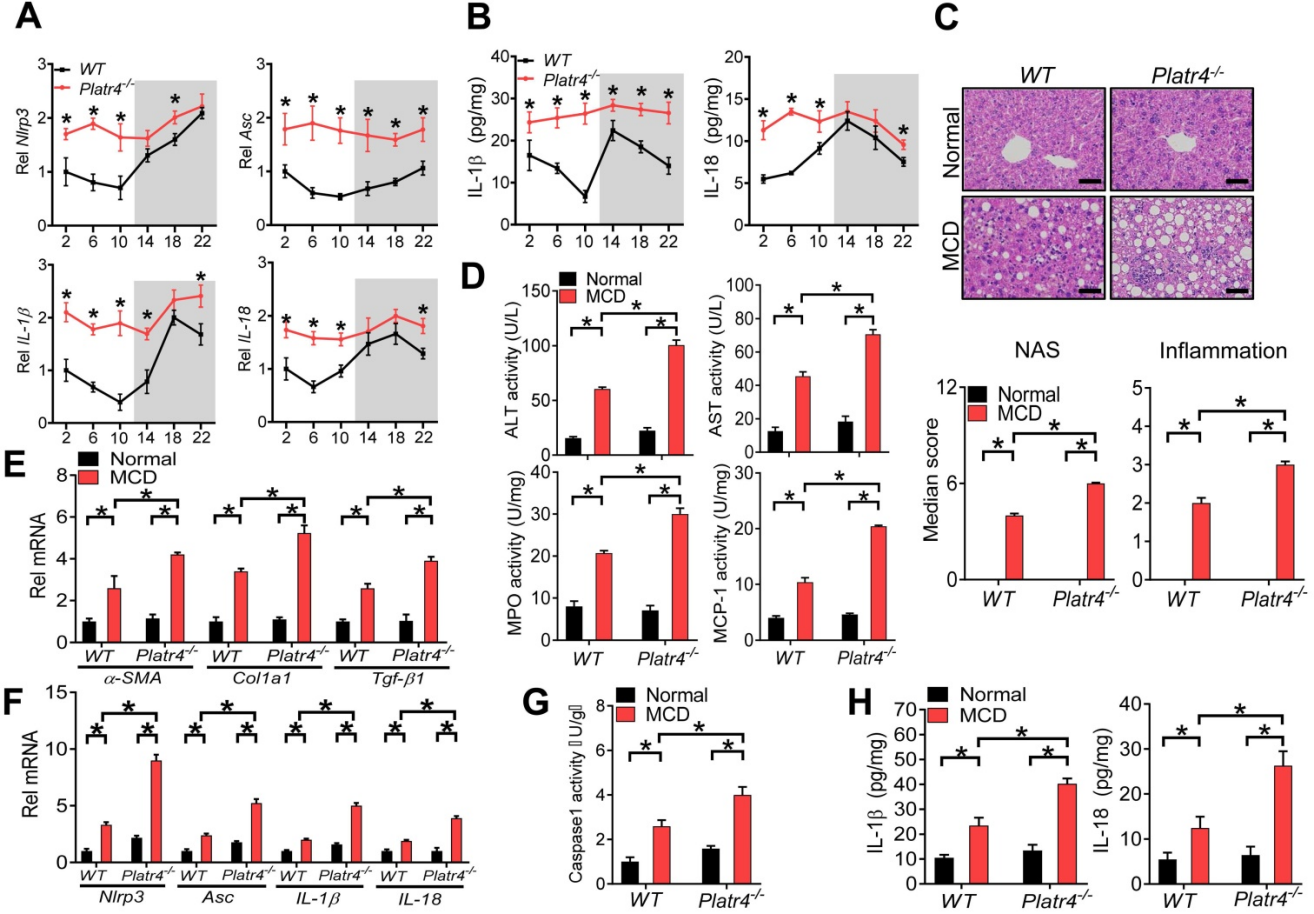
** Loss of *Platr4* blunts the oscillation of *Nlrp3* inflammasome and sensitizes mice to experimental steatohepatitis. (A)** Hepatic *Nlrp3*, *Asc*, *IL-1β* and *IL-18* mRNA levels in wild-type (WT) and *Platr4^-/-^* mice at 6 circadian time points. Data are mean ± SD (*n* = 5). *p <0.05 versus WT at individual time points (two-way ANOVA and Bonferroni post hoc test).** (B)** IL-1β and IL-18 proteins in the livers derived from WT or *Platr4^-/-^* mice at 6 circadian time points. Data are mean ± SD (*n* = 5). *p <0.05 versus WT at individual time points (two-way ANOVA and Bonferroni post hoc test).** (C)** H&E staining of livers from WT and *Platr4^-/-^*mice fed on normal or MCD diet (top panel). Scale bar = 50 μm. NAS and inflammation scores are shown in the bottom panel. *p <0.05 (two-way ANOVA and Bonferroni post hoc test).** (D)** Plasma ALT/AST and hepatic MPO/MCP-1 activities in WT and *Platr4^-/-^*mice fed on normal or MCD diet. Data are mean ± SD (*n* = 8). *p <0.05 (two-way ANOVA and Bonferroni post hoc test).** (E)** Hepatic *α-SMA*, *Col1a1* and *Tgf-β1* mRNAs in WT and *Platr4^-/-^*mice fed on normal or MCD diet. Data are mean ± SD (*n* = 8). *p <0.05 (two-way ANOVA and Bonferroni post hoc test).** (F)** Hepatic *Nlrp3*, *Asc*, *IL-1β* and *IL-18* mRNAs in WT and *Platr4^-/-^*mice fed on normal or MCD diet. Data are mean ± SD (*n* = 8). *p <0.05 (two-way ANOVA and Bonferroni post hoc test).** (G)** Caspase-1 activity in livers of WT and *Platr4^-/-^*mice fed on normal or MCD diet. Data are mean ± SD (*n* = 8). *p <0.05 (two-way ANOVA and Bonferroni post hoc test).** (H)** Hepatic IL-1β (left panel) and IL-18 (right panel) levels in livers of WT and *Platr4^-/-^*mice fed on normal or MCD diet. Data are mean ± SD (*n* = 8). *p <0.05 (two-way ANOVA and Bonferroni post hoc test).

We next tried to determine whether *Platr4* regulates *Nlrp3* inflammasome *in vivo*. To this end, we established *Platr4*-deficient (*Platr4^-/-^*) mice by deleting the two exons of *Platr4* gene (Figure S6). *Platr4 k*nockout did not affect the expression of its neighbor gene *Jade1* (Figure S7)*.* Mouse liver samples were collected every 4-h over a 24-h light-dark cycle to assess the regulatory effects of *Platr4* on *Nlrp3* inflammasome considering that *Nlrp3* inflammasome pathway displays a diurnal rhythm 14. Hepatic* Nlrp3* and *Asc* expression were up-regulated and their rhythms were blunted in *Platr4^-/-^* mice (Figure 4A). Likewise, *IL-1β* and *IL-18* mRNAs were elevated and their oscillations were dampened (Figure 4A). Accordingly, *Platr4* ablation led to increased levels of and altered rhythmicity of produced IL-1β and IL-18 in the liver (Figure 4B). These data indicated that *Platr4* is essential to drive diurnal oscillations in *Nlrp3* inflammasome expression and activity.

*Nlrp3* inflammasome plays a central role in innate immune responses and in development of nonalcoholic steatohepatitis 41. We thus wondered whether deficiency of *Platr4* is associated with exacerbated steatohepatitis. Both *Platr4^-/-^* and wild-type mice were challenged with an MCD diet to induce steatohepatitis. Loss of *Platr4* increased the sensitivity of mice to MCD-induced steatohepatitis as evidenced by a higher overall NAS (NAFLD activity score) value and more severe inflammation in *Platr4^-/-^* mice than in wild-type mice as derived from histopathological examinations (Figure 4C). Supporting this, MCD-challenged *Platr4^-/-^* mice showed higher levels of plasma ALT (alanine aminotransferase) and AST (aspartate aminotransferase) as well as higher activities of liver MPO (myeloperoxidase) and MCP-1 (chemoattractant protein-1) as compared with wild-type mice (Figure 4D). Also, hepatic levels of the pro-fibrotic markers *α-SMA*, *Col1a1* and *Tgf-β1* were higher in *Platr4^-/-^* mice with steatohepatitis than in wild-type controls (Figure 4E). More severe steatohepatitis in *Platr4^-/-^* mice was associated with higher hepatic levels of *Nlrp3*, *Asc*, *IL-1β* and *IL-18* mRNAs, accompanied by an elevated caspase-1 activity and higher levels of IL-1β and IL-18 proteins (Figure 4F-H). Collectively, our data suggested a regulatory role of *Platr4* in experimental steatohepatitis via controlling *Nlrp3* inflammasome activity.

### *Platr4* regulates expression of *Nlrp3* and *Asc* in an NF-κB-dependent manner

**Figure 5:**
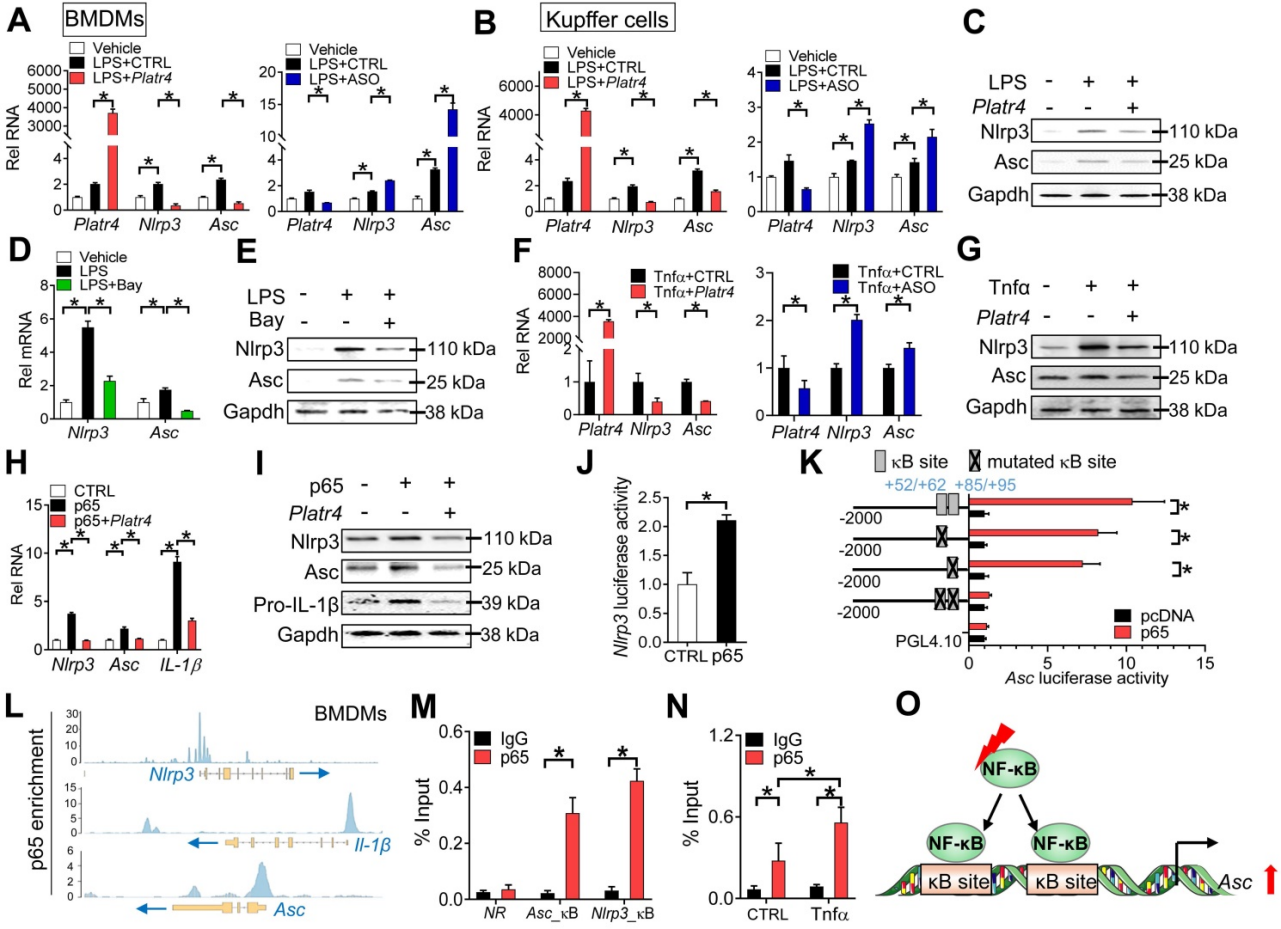
***Platr4* regulates expression of *Nlrp3* and *Asc* in an NF-κB-dependent manner. (A)** Effects of *Platr4* overexpression (left panel) and knockdown (right panel) on *Nlrp3* and *Asc* mRNAs in LPS-stimulated BMDMs. Data are mean ± SD (*n* = 3). *p <0.05 (one-way ANOVA and Bonferroni post hoc test).** (B)** Effects of *Platr4* overexpression (left panel) and knockdown (right panel) on *Nlrp3* and *Asc* mRNAs in LPS-stimulated Kupffer cells. Data are mean ± SD (*n* = 3). *p <0.05 (one-way ANOVA and Bonferroni post hoc test).** (C)** Effects of *Platr4* overexpression on *Nlrp3* and *Asc* proteins in LPS-stimulated BMDMs. **(D)** Effects of Bay 11-7082 (Bay, 5 μM) on *Nlrp3* and *Asc* mRNAs in LPS-stimulated BMDMs. Data are mean ± SD (*n* = 3). *p <0.05 (one-way ANOVA and Bonferroni post hoc test).** (E)** Effects of Bay 11-7082 (5 μM) on *Nlrp3* and *Asc* proteins in LPS-stimulated BMDMs. Data are mean ± SD (*n* = 3).** (F)** Effects of *Platr4* overexpression (left panel) and knockdown (right panel) on *Nlrp3* and *Asc* mRNAs in Tnfα-stimulated BMDMs. Data are mean ± SD (*n* = 3). *p <0.05 (t-test).** (G)** Effects of *Platr4* overexpression on *Nlrp3* and *Asc* proteins in Tnfα-stimulated BMDMs. **(H)** Effects of *Platr4* on *Nlrp3* and *Asc* mRNAs in p65-overexpressed BMDMs. Data are mean ± SD (*n* = 3). *p <0.05 (one-way ANOVA and Bonferroni post hoc test).** (I)** Effects of *Platr4* on *Nlrp3* and *Asc* proteins in p65-overexpressed BMDMs. **(J)** p65 activates *Nlrp3* transcription in luciferase reporter assay with BMDMs. Data are mean ± SD (*n* = 6). *p <0.05 (t-test).** (K)** Effects of p65 on the activities of various versions of *Asc-Luc* reporters in BMDMs. Data are mean ± SD (*n* = 6). *p <0.05 (t-test).** (L)** p65 binding peaks at the *Nlrp3*, *IL-1β*, and *Asc* promoters derived from published ChIP-seq data (GSM2522473). **(M)** ChIP assays showing recruitment of p65 protein to the κB sites of *Asc* promoter in mouse liver. Data are mean ± SD (*n* = 3). *p <0.05 (t-test). NR, non-binding region. **(N)** Effects of Tnfα (20 ng/ml) on recruitment of p65 protein to *Asc* promoter. Data are mean ± SD (*n* = 3). *p <0.05 (two-way ANOVA and Bonferroni post hoc test).** (O)** Schematic diagram showing transcriptional regulation of *Asc* by NF-κB.

NF-κB is a known upstream transcriptional activator of *Nlrp3* and a potential regulator of *Asc* 42,43. We next investigated whether NF-κB plays a role in regulation of *Nlrp3* and *Asc* by *Platr4*.* Platr4* overexpression decreased, whereas *Platr4* knockdown increased, the mRNA levels of both *Nlrp3* and *Asc* in LPS-stimulated BMDMs and Kupffer cells (Figure 5A-B). The alterations in* Nlrp3* and *Asc* mRNAs paralleled those in their proteins (Figure 5C), suggesting involvement of transcriptional repression in* Platr4*-mediated regulation. Bay 11-7082 (a specific inhibitor of NF-κB) attenuated the mRNA and protein expressions of both *Nlrp3* and *Asc* in LPS-stimulated BMDMs (Figure 5D-E). Tnfα is an activator of NF-κB signaling pathway 44. Overexpression of* Platr4* inhibited, whereas knockdown of *Platr4* increased, the expression levels of *Nlrp3* and *Asc* in Tnfα-treated BMDMs (Figure 5F-G). Furthermore, overexpression of p65 (an NF-κB subunit) induced the expression of *Nlrp3* and *Asc* (Figure 5H-I). However, the induction effects of p65 were diminished in the presence of *Platr4* (Figure 5H-I). These findings supported NF-κB-dependent regulation of *Nlrp3* and *Asc* by *Platr4*. *Nlrp3* has been previously shown to be a direct target gene of NF-κB 45. We confirmed that NF-κB p65 indeed activated *Nlrp3* transcription (Figure 5J). We further tested whether NF-κB directly regulated *Asc* gene transcription. In luciferase reporter assay, p65 induced the transcriptional activity of *Asc* (Figure 5K). Promoter analysis identified two potential κB sites (NF-κB-binding sites, located at +52/+62 bp and +85/+95 bp, respectively) in *Asc* promoter. The activation effect of p65 was attenuated when single site was mutated, and was completely lost when both sites were mutated (Figure 5K). Supporting this, p65 protein was significantly recruited to the κB sites of* Asc* according to ChIP-seq analysis and ChIP assays (Figure 5L-M), and the recruitment was enhanced in the presence of Tnfα (Figure 5N). Therefore, NF-κB trans-activated *Asc* via direct binding to two κB sites in gene promoter (Figure 5O). Altogether, regulation of *Nlrp3* and *Asc* expression by *Platr4* was probably through inhibition of their transcriptional activator NF-κB.

### *Platr4* inhibits NF-κB activity via interacting with Rxrα

**Figure 6:**
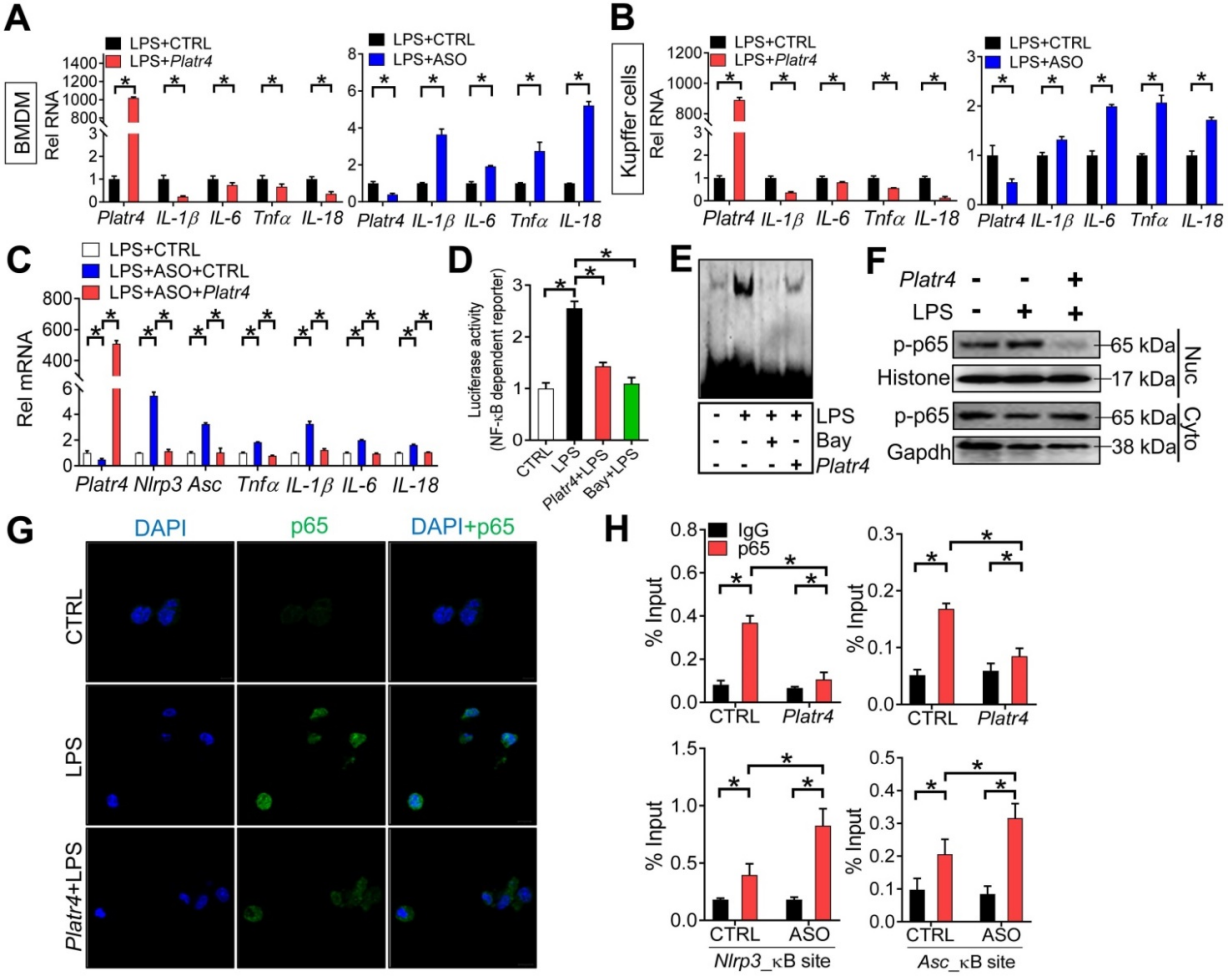
***Platr4* inhibits NF-κB activity. (A)** Effects of *Platr4* overexpression and knockdown on NF-κB target genes (*IL-1β, IL-6, Tnfα* and *IL-18*) in LPS-stimulated BMDMs. Data are mean ± SD (*n* = 3). *p <0.05 (t-test).** (B)** Effects of *Platr4* overexpression and knockdown on NF-κB target genes (*IL-1β, IL-6, Tnfα* and *IL-18*) in LPS-stimulated Kupffer cells. Data are mean ± SD (*n* = 3). *p <0.05 (t-test).** (C)** Rescue experiments showing that *Platr4* knockdown-induced changes in expressions of NF-κB target genes can be restored by *Platr4* overexpression. BMDMs were transfected with ASO or negative control. 48 h later, the medium was replaced with fresh medium and BMDMs were transfected with *Platr4* or pcDNA blank plasmid for 24 h. Cells were collected for qPCR assays. Data are mean ± SD (*n* = 3). *p <0.05 (one-way ANOVA and Bonferroni post hoc test).** (D)** Effects of* Platr4* and Bay (Bay 11-7082, 5 μM) on NF-κB-dependent reporter activity in LPS-stimulated BMDMs. Data are mean ± SD (*n* = 6). *p <0.05 (one-way ANOVA and Bonferroni post hoc test).** (E)** Effects of *Platr4* and Bay (5 μM) on binding of NF-κB DNA probe to nuclear proteins derived from LPS-stimulated BMDMs. **(F)** Effects of *Platr4* on the level of phosphorylated (p)-p65 in nuclear (Nuc) and cytoplasmic (Cyto) fractions of LPS-stimulated BMDMs. **(G)** Immunofluorescence images showing that *Platr4* suppresses nuclear translocation of p65 in LPS-stimulated BMDMs. **(H)** ChIP assays showing that *Platr4* overexpression reduces, whereas *Platr4* knockdown enhances, the recruitment of p65 protein to κB sites in *Nlrp3* and *Asc* promoters. Data are mean ± SD (*n* = 3). *p <0.05 (two-way ANOVA and Bonferroni post hoc test).

We next tested whether *Platr4* affects the activity of NF-κB. Overexpression of *Platr4* decreased the mRNA levels of known NF-κB target genes (e.g., *IL-1β*, *IL-6*, *Tnfα* and *IL-18*) in LPS-stimulated BMDMs and Kupffer cells, while *Platr4* knockdown increased the expressions of these genes (Figure 6A-B) 46. *Platr4* knockdown-induced changes in expressions of NF-κB target genes (i.e., *IL-1β*, *IL-6*, *Tnfα*, *IL-18*, *Nlrp3* and *Asc*) can be restored by *Platr4* overexpression, confirming a specific inhibitory effect of *Platr4* on NF-κB activity (Figure 6C). Supporting this, *Platr4* inhibited the transcriptional activity of NF-κB according to the luciferase reporter and EMSA assays, similar effects as observed for the specific NF-κB inhibitor Bay 11-7082 (Figure 6D-E). Phosphorylation of p65 is implicated in optimal NF-κB activation and in controlling NF-κB directed transactivation 47. We found that *Platr4* was able to decrease the levels of both phosphorylated p65 (p-p65) and p65 (measured by immunofluorescence analysis) in the nuclei (Figure 6F-G). Moreover, recruitment of p65 onto the promoters of NF-κB target genes (*Nlrp3* and* Asc*) was inhibited by overexpression of *Platr4*, while *Platr4* knockdown led to enhanced recruitment of p65 (Figure 6H) 48. Overall, these findings indicated that *Platr4* inhibits NF-κB activity via reducing NF-κB binding to target genes.

**Figure 7:**
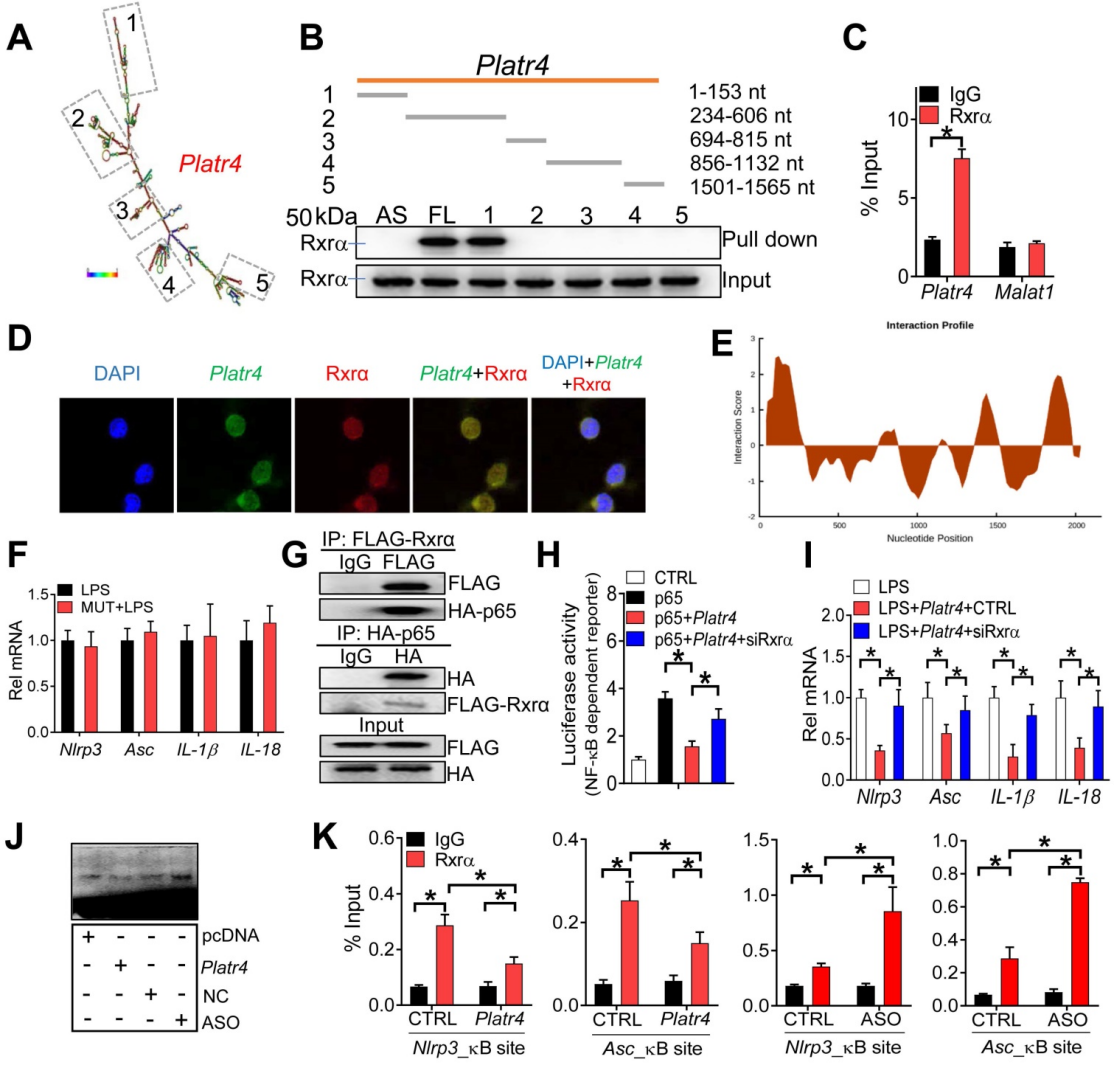
***Platr4* interacts with Rxrα to modulate the activity of NF-κB. (A)** Secondary structure for *Platr4* with minimum free energy predicted by Vienna RNA web server.** (B)** Western blotting analyses of interactions of Rxrα protein with various versions of* Platr4* probes following RNA-pull down assays. Full-length (FL), antisense (AS) and truncated fragments are labeled.** (C)** RIP assay showing an interaction between Rxrα protein and *Platr4* in mouse liver. Data are mean ± SD (*n* = 3). *p <0.05 (t-test).** (D)** Immunofluorescence assays showing colocalization of* Platr4* and Rxrα protein in BMDMs.** (E)** Prediction of interaction propensity between Rxra and full length *Platr4* (catRAPID). Positive interaction score predicts increased propensity of binding. **(F)** Effects of a *Platr4* mutant (1-153 nt fragment was mutated) on expression of NF-κB target genes (*Nlrp3, Asc, IL-1β* and *IL-18*) in LPS-stimulated BMDMs. Data are mean ± SD (*n* = 3). **(G)** Co-IP assays showing an interaction between Rxrα and p65 proteins. **(H)** Effects of Rxrα knockdown on the *Platr4*-mediated inhibition of* NF-κB* reporter activity in p65-overexpressed BMDMs. Data are mean ± SD (*n* = 6). *p <0.05 (one-way ANOVA and Bonferroni post hoc test).** (I)** Effects of Rxrα knockdown on *Platr4*-mediated inhibition of* Nlrp3*, *Asc*, *IL-1β* and *IL-18* mRNAs in LPS-stimulated BMDMs. Data are mean ± SD (*n* = 3). *p <0.05 (one-way ANOVA and Bonferroni post hoc test).** (J)** Effects of *Platr4* overexpression and knockdown on binding of NF-κB to its DNA probe in Rxrα- and p65- ovexpressed BMDMs.** (K)** ChIP assays showing that Rxrα protein is recruited to the κB sites of *Nlrp3* and *Asc* promoters, and that the recruitment is inhibited by overexpression of *Platr4* but enhanced when *Platr4* is knocked-down. Data are mean ± SD (*n* = 3). *p <0.05 (two-way ANOVA and Bonferroni post hoc test).

Nuclear lncRNAs may interact with target proteins to regulate their functions 49. *Platr4* may be one of such lncRNAs due to the presence of five stem loop structures (i.e., 1-153 nt, 234-606 nt, 694-815 nt, 856-1132 nt and 1501-1565 nt) (Figure 7A). RNA pull-down assays followed by mass spectrometric analysis and Western blotting identified retinoid X receptor α (Rxrα, ~50 kDa) as a nuclear protein interacting with* Platr4* in mouse liver (Figure 7B and Table S1). A direct interaction of *Platr4*, but not *Malat1* (another lncRNA mainly distributed in the nucleus), with Rxrα was further confirmed by a RIP (RNA immunoprecipitation) assay (Figure 7C). Moreover, *Platr4* and Rxrα co-localized in the nuclei of BMDMs (Figure 7D). Truncation analyses indicated that the fragment of 1-153 nt was responsible for Rxrα binding (Figure 7B), that agreed with the highest predicted interaction score for this fragment (Figure 7E). Supporting this, *Platr4* carrying a mutation of 1-153 nt fragment failed to inhibit the expressions of NF-κB target genes (i.e., *Nlrp3*, *Asc, IL-1β* and *IL-18*) in LPS-stimulated BMDMs (Figure 7F).

NF-κB constitutively binds to RXR receptor through the N-terminal ABC domains of RXR and the Rel homology domains of p50 and p65 23. The NF-κB/RXR complex is κB site-binding competent and transcriptionally active 23. We confirmed a physical interaction between murine Rxrα and p65 based on Co-IP (co-immunoprecipitation) assays (Figure 7G). Knockdown of Rxrα decreased the expression levels of* Nlrp3* and other NF-κB target genes in LPS-stimulated BMDMs (Figure S8). Also, Rxrα enhanced the promoter activity of an NF-κB reporter in BMDMs stimulated with Tnfα, and promoted the promoter activity of *Nlrp3* reporter in the presence or absence of p65 (Figure S9). Moreover, Rxrα knockdown was associated with reduced recruitment of p65 to the κB sites of *Nlrp3* and *mCSF* (a known NF-κB target gene) (Figure S10). All these supported a role of Rxrα in regulating NF-κB activity via a physical interaction.

Since Rxrα interacts with both *Platr4* and NF-κB, we thus examined whether *Platr4* regulates NF-κB activity through Rxrα. The effects of *Platr4* on NF-κB-dependent reporter activity and NF-κB target genes (i.e., *Nlrp3* and *Asc*) were attenuated when *Rxrα* was silenced (Figure 7H-I). Furthermore, according to the EMSA assays, overexpression of *Platr4* inhibited, while *Platr4* knockdown enhanced, the DNA-binding activity of NF-κB in the presence of Rxrα (Figure 7J). ChIP assays showed significant recruitment of both Rxrα and p65 to the κB sites of *Nlrp3* and *Asc* promoters (Figure 6H-7K). Recruitment of these two proteins was inhibited by overexpression of *Platr4*, but enhanced when *Platr4* was knocked-down (Figure 6H-7K). Additionally, we observed unaffected expression of Rxrα in *Platr4* overexpressed or knocked-down cells (Figure S11). These findings indicated that *Platr4* prevents binding of the NF-κB/Rxrα complex to κB sites, thereby inhibiting the transactivation activity of NF-κB. By referring to the main archetypes of lncRNA mechanisms 49, we postulated that *Platr4* may act as a decoy and titrate away Rxrα and its partner NF-κB from the promoters of target genes such as* Nlrp3* and *Asc*.

### Overexpression of *Platr4* ameliorates experimental steatohepatitis in an *Nlrp3*-dependent manner

**Figure 8:**
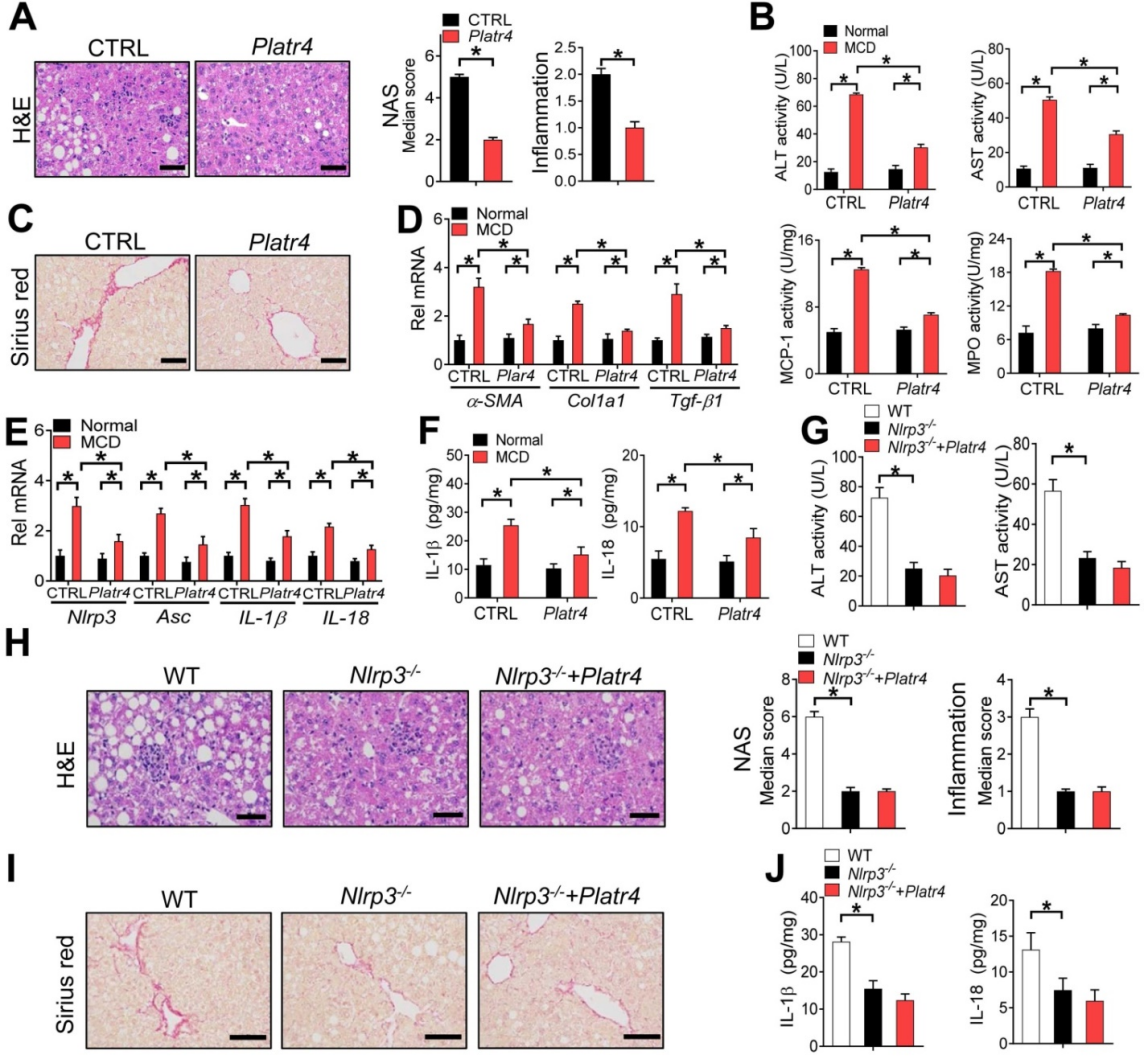
** Overexpression of *Platr4* ameliorates experimental steatohepatitis in an *Nlrp3*-dependent manner. (A)** H&E staining of livers from *Platr4*-overexpressed (AAV8.TBG.*Platr4*) and control mice fed on an MCD diet (left panel). Scale bar = 50 μm. NAS and inflammation scores are shown in the right panel. Data are mean ± SD (*n* = 8). * *p <*0.05 (t-test).** (B)** Plasma ALT/AST and hepatic MPO/MCP-1 activities in *Platr4*-overexpressed and control mice fed on normal or MCD diet. Data are mean ± SD (*n* = 8). *p <0.05 (two-way ANOVA and Bonferroni post hoc test).** (C)** Sirius red staining of livers from *Platr4*-overexpressed and control mice fed on a MCD diet. Scale bar = 50 μm.** (D)** Hepatic *α-SMA*, *Col1a1* and *Tgf-β1* mRNAs in *Platr4*-overexpressed and control mice fed on normal or MCD diet. Data are mean ± SD (*n* = 8). *p <0.05 (two-way ANOVA and Bonferroni post hoc test).** (E)** Hepatic *Nlrp3*, *Asc*, *IL-1β* and *IL-18* mRNAs in *Platr4*-overexpressed and control mice fed on normal or MCD diet. Data are mean ± SD (*n* = 8). *p <0.05 (two-way ANOVA and Bonferroni post hoc test).** (F)** IL-1β and IL-18 in livers of *Platr4*-overexpressed and control mice fed on normal or MCD diet. Data are mean ± SD (*n* = 8). *p <0.05 (two-way ANOVA and Bonferroni post hoc test).** (G)** Plasma ALT and AST activities in *Platr4*-overexpressed* Nlrp3^-/-^* mice and control mice fed on a MCD diet. Data are mean ± SD (*n* = 8). *p <0.05 (one-way ANOVA with Bonferroni post hoc test). **(H)** H&E staining of livers from *Platr4*-overexpressed* Nlrp3^-/-^* mice and control mice fed on a MCD diet. Scale bar = 50 μm. NAS and inflammation scores are shown in the rightmost panel. Data are mean ± SD (*n* = 8). *p <0.05 (one-way ANOVA with Bonferroni post hoc test). **(I)** Sirius red staining of livers from *Platr4*-overexpressed* Nlrp3^-/-^* mice and control mice fed a MCD diet. Scale bar = 50 μm. **(J)** Hepatic *IL-1β* and *IL-18* levels in *Platr4*-overexpressed* Nlrp3^-/-^* mice and control mice fed on an MCD diet. Data are mean ± SD (*n* = 8). *p <0.05 (one-way ANOVA with Bonferroni post hoc test).

The role of *Platr4* in inhibition of *Nlrp3* inflammasome prompted us to test whether targeted overexpression of *Platr4* in the liver can prevent steatohepatitis. We thus generated a recombinant adeno-associated virus serotype 8 (AAV8) vector encoding *Platr4* driven by a liver-specific thyroxine-binding globulin (TBG) promoter (named “AAV8.TBG.*Platr4*”). AAV8.TBG.*Platr4* was able to increase hepatic expression of *Platr4* by over 100-fold on day 15 after a single vector injection (Figure S12). According to histopathological examinations, overexpression of *Platr4* protected mice from MCD-induced steatohepatitis because of lower NAS and inflammation scores in AAV8.TBG.*Platr4*-treated mice than in control mice (Figure 8A). Supporting this, MCD-challenged and AAV8.TBG.*Platr4*-treated mice showed lower levels of plasma ALT and AST as well as reduced activities of liver MPO and MCP-1 as compared with control mice (Figure 8B). Overexpression of *Platr4* also retarded the development of liver fibrosis in steatohepatitis mice as revealed by the Sirius red staining (Figure 8C). This was in accordance with lower hepatic levels of the pro-fibrotic markers *α-SMA*, *Col1a1* and *Tgf-β1* in *Platr4*-overexpressed mice (Figure 8D). Intriguingly, alleviation of steatohepatitis in *Platr4*-overexpressed mice was associated with lower hepatic levels of *Nlrp3*, *Asc*, *IL-1β* and *IL18* mRNAs, and with reduced production of IL-1β and IL18 proteins, suggesting a role of *Nlrp3* inflammasome inactivation in *Platr4* prevention of steatohepatitis (Figure 8E-F). To corroborate this finding, we determined the effects of *Platr4* overexpression on steatohepatitis development in *Nlrp3*-deficient (*Nlrp3^-/-^*) mice. In line with previous reports, *Nlrp3* ablation in mice reduced the severity of MCD-induced steatohepatitis 12. The protective effect of *Nlrp3* ablation on disease was similar to that elicited by *Platr4* overexpression in wild-type mice (Figure 8G-J). However, *Platr4* overexpression showed no effects on steatohepatitis development in *Nlrp3^-/-^* mice (Figure 8G-J). Altogether, our data indicated that targeted overexpression of *Platr4* in the liver ameliorates experimental steatohepatitis in an *Nlrp3*-dependent manner.

## Discussion

It is imperative to understand the pathogenesis of NALFD and NASH and to search for specific treatments due to a high incidence and prevalence. Given that the *NLRP3* inflammasome plays a central role in chronic inflammatory diseases such as NASH and displays a circadian rhythmicity, it was hypothesized that *NLRP3* inflammasome and steatohepatitis may be regulated by an oscillating lncRNA (i.e., rhythmically expressed). In an attempt to identify a potential oscillating lncRNA associated with steatohepatitis, we applied two screening criteria, namely, steatohepatitis-regulated and Rev-erbα (a core clock component)-controlled lncRNA (Figures 1-2). *Platr4* with a liver-specific expression was screened as a cycling lncRNA that up-regulated the most in mice with MCD-induced steatohepatitis. This lncRNA was initially identified in an RNA-seq screen of embryonic stem cells but the exact functions remain underexplored 32. We have further defined an anti-*Nlrp3* inflammasome function of *Platr4* and established a negative relationship between the abundance of* Platr4* and the severity of experimental steatohepatitis. Therefore, *Platr4* may be an attractive target for nonalcoholic steatohepatitis. However, there is a limitation for the translational potential because it is necessary to increase the expression of this lncRNA for its effect, which is not the common way the therapeutic approaches are currently addressing.

Our findings may suggest a self-protection mechanism against steatohepatitis, which involves auto-inhibition of NF-κB. Activated NF-κB pathway in steatohepatitis transcriptionally drives the expression of *Platr4* that in turn inhibits NF-κB activity and inactivates *Nlrp3* inflammasome by preventing binding of NF-κB to κB sites in the promoters of target genes including *Nlrp3* and *Asc*. Mechanistically, *Platr4* physically interacts with Rxrα protein and titrates it and its constitutive partner NF-κB p65 away from the κB binding site, thereby reducing the transcriptional activity of NF-κB and suppressing *Nlrp3* inflammasome activation. This conforms to a general RNA-protein interaction mechanism for lncRNA actions 24. We argue that *Platr4* may bring the NF-κB/Rxrα complex away from the nucleus (probably directed to the cytoplasm) because of a reduction in nuclear accumulation of NF-κB in the presence of *Platr4*, although the mechanisms underlying this process remain to be determined (Figure 6). The proposed self-protection mechanism may support the notion that the body has certain ability to self-heal diseases 50. However, the protective effect of *Platr4* may be overwhelmed by MCD-induced inflammation insult. In other words, *Platr4* (a several-fold increase) may be insufficient to reverse the massive and destructive inflammatory responses caused by MCD feeding, otherwise, MCD cannot induce steatohepatitis at all.

Circadian gene expression is mainly driven by the molecular components of circadian clock via direct transcriptional actions on their *cis-*elements (E-box, D-box and RevRE or RORE) 35,36. Alternatively, an indirect mechanism has been proposed for generation of rhythmic gene expression by circadian clock 35. This mechanism involves cycling transcriptional factors (TFs) (including nuclear receptors) as the intermediates. The rhythms of clock-output cycling TFs are propagated to their downstream target genes. Examples of such cycling TFs are aryl hydrocarbon receptor, peroxisome proliferator-activated receptor gamma (Ppar-γ), hepatocyte nuclear factor 4α (Hnf4α), and Hnf1α 35,51. Our finding that *Platr4* regulates the circadian rhythmicity in *Nlrp3* inflammasome pathway herein leads to a proposal of an additional mechanism for circadian regulation of immune genes. That is rhythmic gene expression can be partially accounted for by clock-controlled oscillating lncRNAs. This type of lncRNAs periodically interact with target proteins (or other molecules), thereby generating rhythms in transcriptional activities and in target gene expression.

We have revealed a physical interaction between *Platr4* and Rxrα in the liver, that leads to a reduction in the transcriptional activity of NF-κB as an Rxrα heterodimer partner. It is well-known that many NRs (e.g., constitutive androstane receptor, pregnane X receptor, farnesoid X receptor, vitamin D receptor, and PPARs) function as a heterodimer with RXR. This raises a possibility that *Platr4* may modulate the transcriptional activities of these RXR-interacting NRs in a similar manner as it does to the NF-κB. The functions of *Platr4* (as a NR modifier) thus may be extended to many other aspects of biology and physiology. It is noteworthy that the transcriptional activity (for NF-κB) of the NF-κB/RXR complex depends on the RXR ligands (e.g., retinoids) 23. In the absence of retinoids, the complex is transcriptionally active and favorable as evidenced by the observations that (1) overexpression of Rxrα increases, whereas knockdown of Rxrα decreases, the expression levels of* Nlrp3* and other NF-κB target genes (Figure S8); (2) Rxrα enhances the promoter activity of an *NF-κB* reporter in macrophages, and promotes the promoter activity of *Nlrp3* reporter in the presence or absence of p65 (Figure S9); and (3) Rxrα knockdown was associated with reduced recruitment of p65 to the κB sites of *Nlrp3* (Figure S10). However, the protein complex becomes inactive in the presence of a retinoid 23,52. Na and coworkers propose that retinoid binding may induce conformational changes in RXR protein, leading to inhibition of the interactions between NF-κB and κB site 23. Alternatively, co-activators (e.g., SRC-1 and CBP/p300) may constitutively bind NF-κB but recognizes RXR only in the presence of a retinoid 23.

We have demonstrated that *Platr4* inactivates *Nlrp3* inflammasome via inhibiting the transcription of both *Nlrp3* and* Asc* in an NF-κB-dependent manner. It is a previously unreported finding that NF-κB trans-activates* Asc* through direct binding to two κB sites (i.e., +52/+62 bp and +85/+95 bp) in the gene promoter (Figure 5). Our finding sheds light on previously unexplained observations that *ASC* is up-regulated by inflammation in human neutrophils, and that *ASC* is up-regulated by TNFα in breast epithelial cells and the induction effect is attenuated when p65 is knocked-down 43,53. Contrasting with the general notion that the purpose of inflammasome priming is to increase the expression levels of *Nlrp3* and IL-1β via NF-κB activation 9, inflammasome activation may also require an increase in *ASC* expression occurring in the priming stage.

The lncRNA (*Platr4*) may represent a new layer of molecular mechanisms for the regulation of inflammation by circadian clock. Prior studies have indicated that regulation of inflammation is attained through the core components of circadian clock such as Clock, Rev-erbα and Cry1/2, accounting for diurnal rhythmicity in the severity (symptom) of inflammatory diseases (e.g., colitis and rheumatoid arthritis) and increased susceptibility to inflammation-related diseases (e.g., obesity and NAFLD) 54-59. *Platr4* appears to act as an integrator of circadian clock and inflammation through the NF-κB/*Nlrp3* inflammasome axis. Defining this integrator role for *Platr4* enhances our understanding of the crosstalk between circadian clock and inflammation, and highlights a complexity in circadian modifiers of inflammation. We argue for an essential role of potential alternative factors beyond the clock components in controlling the rhythmicity of *Nlrp3* inflammasome pathway because loss of Rev-erbα fails to cause elevations over the entire light/dark cycle in *Nlrp3* expression as observed for a typical target gene such as *Bmal1* 14,54,60. We propose that* Platr4* may act as one of such “alternative factors” in the liver that has a significant contributing role in circadian behaviors of hepatic inflammation (Figure 4). Regulation by oscillating *Platr4* (as an NF-κB repressor) may contribute to previously reported diurnal variations in transcriptional activity of NF-κB with a zenith at ZT6 that corresponds to the trough expression of *Platr4* 57.

MCD diet model was selected herein to study the regulation mechanisms of *Nlrp3* inflammasome and steatohepatitis as noted in prior studies 12,61. The deficiency in choline and methionine, two essential nutrients, results in impaired β-oxidation and compromised production and secretion of very low-density lipoprotein particles, and therefore in hepatic fat accumulation, ballooning, inflammation and early development of fibrosis, which are typical characteristics of steatohepatitis 62. The MCD model is considered adequate to study the intrahepatic events in relation to steatohepatitis and the therapeutic interventions 62. However, this model may not reflect the metabolic changes (e.g., insulin resistance and obesity) associated with typical human steatohepatitis 62.

It is our future work to identify the equivalent lncRNA of *Platr4* in humans. It may be not a practical approach using evolutional history to predict a function of lncRNA in different species because the current comparative genomic tools cannot easily detect homologs among lncRNAs from different species 63,64. Therefore, it remains a challenge to identify a human “homolog” of* Platr4*, which has a similar structure or function to mouse *Platr4*. Considering this, we plan to use anti-RXRα antibody to reciprocally pull-down the lncRNAs that are associated with RXRα in livers from human samples. By using RNA-seq techniques, we would identify the lncRNAs that might modulate RXRα function and examine their functions in regulation of hepatic protein turnover.

In summary, the oscillating lncRNA *Platr4* functions as a circadian repressor of *Nlrp3* inflammasome by inhibiting NF-κB-dependent transcription of *Nlrp3* and Asc. Mechanistically, *Platr4* prevents binding of the NF-κB/Rxrα complex to κB sites via a physical interaction. Accordingly, loss of *Platr4* sensitizes mice to experimental steatohepatitis, whereas overexpression of *Platr4* ameliorates the pathological conditions. Therefore, *Platr4* may be an attractive target that can be modulated to ameliorate the pathological conditions of steatohepatitis.

## Methods

### Materials

Murine macrophage colony-stimulating factor (M-CSF) was purchased from Peprotech (Rocky Hill, NJ). Pam3CSK4 was purchased from InvivoGen (San Diego, CA). Poly (I:C), nigericin, LPS and collagenase IV were purchased from Sigma-Aldrich (St. Louis, MO). Pronase E was purchased from Yuanye Bio-Technology Company (Shanghai, China). ChIP kit was purchased from Cell Signaling Technology (Beverly, MA). Bay 11-7082, murine Tnfα, 4',6-diamidino-2-phenylindole (DAPI), BCA assay kit, cytoplasmic/nuclear protein extraction kit and EMSA kit were purchased from Beyotime (Shanghai, China). Cytoplasmic/nuclear RNA purification kit was purchased from Norgen Biotek (Belmont, CA). InRcute lncRNA First-Strand cDNA Synthesis kit (with gDNase) and FastQuant RT kit (with gDNase) were purchased from Tiangen (Beijing, China). RNAiso Plus reagent and PrimeScript RT Master Mix were purchased from Vazyme (Nanjing, China). JetPrime transfection kit was purchased from POLYPLUS Transfection (Ill kirch, France). Anti-*Nlrp3*, anti-caspase-1 and anti-*Asc* antibodies were purchased from AdipoGen (San Diego, CA). Anti-IL-1β antibody was purchased from R&D systems (Minneapolis, MN). Anti-p65, anti-Rev-erbα, anti-p-p65 and anti-rabbit IgG antibodies were obtained from Cell Signaling Technology (Danvers, MA). Anti-Gapdh antibody was purchased from Abcam (Cambridge, UK). Anti-Rxrα, anti-FLAG and anti-HA antibodies were obtained from Proteintech Group (Chicago, IL). *Asc* luciferase reporters (-2000/+100 bp and κB site-mutated versions), *Platr4* luciferase reporters (-2000/+100 bp, -1000/+100 bp, -400/+100 bp, and κB or RevRE site-mutated versions), *Nlrp3* luciferase reporter (2.1 kb), pcDNA3.1, pcDNA3.1-p65, pcDNA3.1-Rev-erbα, pcDNA3.1-*Platr4* (the *Platr4*-203 isoform, transcript ID: ENSMUST00000199155.1), siRev-erbα, siBmal1, siRxrα and ASO [targeting the sequenece (GCACUGAGCCAUCUUACUUG) in *Platr4*-203] were obtained from Transheep Technologies (Shanghai, China). *Platr4* probes for RNA pull-down were purchased from GenePharma (Shanghai, China). FISH probe for *Platr4* was obtained from BersinBio (Guangzhou, China). The sequences of siRNAs and ASOs are shown in Table S2.

### Animals

Wild-type C57BL/6 mice were obtained from HFK Bio-Technology (Beijing, China). *Rev-erbα^-/-^*, *Bmal1^-/-^* and *Nlrp3^-/-^* mice (a C57BL/6 background) have been established and validated in our laboratory 54,65,66. *E4bp4^-/-^* mice (a C57BL/6 background) was obtained from Dr. Masato Kubo at RIKEN Institute in Japan 67. *Platr4^-/-^* mice (on a C57BL/6 background) were generated by deleting the DNA fragment (exons 1 and 2) of *Platr4* gene and using the CRISPR/Cas9 technique with the aid of Cyagen Biosciences Inc (Guangzhou, China). All mice were maintained under a 12-h light/12-h dark cycle [lights on at 7 AM (= ZT0) and lights off at 7 PM (= ZT12)] and fed *ad libitum*. For characterization of diurnal gene expression, wild-type and gene knockout (*Rev-erbα^-/-^*, *Bmal1^-/--^* and* E4bp4^-/-^*) mice (male, aged 8-10 weeks) were sacrificed at a 4-h interval around the clock (ZT2, ZT6, ZT10, ZT14, ZT18 and ZT22). Liver samples were collected and snap frozen in liquid nitrogen, and stored at -80 °C until use.

### Steatohepatitis model

Wild-type and gene knockout (*Platr4^-/-^* and* Nlrp3^-/-^*) mice (male, aged 8-10 weeks) were fed a methionine-choline-deficient (MCD) diet (A02082002B, Research Diets) to induce steatohepatitis as previously described 68*.* Control groups of mice were fed a normal diet. After 6 weeks, mice were sacrificed at ZT2, and blood and liver samples were collected for biochemical, expression and/or histological analyses. To evaluate the effects of *Platr4* overexpression on steatohepatitis, wild-type and *Nlrp3^-/-^* mice were pretreated with AAV8.TBG.*Platr4* (2.5×10^11^ virus particles per mouse, Transheep Technologies, Shanghai, China) or control vector via the tail vein injection at ZT10. On day 15, mice were fed an MCD diet to induce steatohepatitis. Six weeks later, mice were sacrificed, and blood and liver samples were collected for biochemical, expression and/or histological analyses.

### Histological analysis

Formalin-fixed liver samples were embedded in paraffin and cut into 4-μm-thick sections. The liver sections were stained with hematoxylin and eosin (H&E). The NAFLD activity score (NAS) were derived based on the severity of hepatic steatosis (with a score of 0-3), lobular inflammation (with a score of 0-3), and hepatocellular ballooning (with a score of 0-2) 69. For assessment of lipid accumulation, 10-μm paraffin sections of liver samples were stained with Oil Red O and counterstained with hematoxylin. Additionally, liver paraffin-sections (5 µm in thickness) were stained with Sirius Red to visualize the collagen content.

### Biochemical analysis

Plasma ALT/AST and hepatic triglyceride levels were measured using enzymatic assay kits (Jiancheng Bioengineering Institute, Nanjing, China). Hepatic IL-1β, IL-6, Tnfα and IL-18 levels, and caspase 1/MCP-1/MPO activities were measured using enzyme linked immunosorbent assay kits (Neobioscience, Shenzhen, China).

### Isolation of BMDMs

BMDMs were isolated from mice as previously described 54. In brief, tibias and femurs were disinfected using 75% ethanol. Bone marrow cavities were rinsed with RPMI 1640 medium. The rinsing solution was collected and centrifuged at 1,000 rpm for 5 min. The pellet cells (BMDMs) were collected and cultured in RPMI 1640 medium containing 10% FBS, 1% penicillin-streptomycin and 20 ng/ml M-CSF. After 7 days, BMDMs were washed using sterile PBS and used for further experiments.

### Isolation of Kupffer cells

Kupffer cells were isolated from mice as previously described 70. In brief, livers were perfused with saline solution for 10 min followed by* in vivo* digestion with liberase enzyme for 5 min and *in vitro* digestion for 30 min. The nonhepatocyte content was subjected to Percoll gradient centrifugation. The intercushion fraction was washed and adhered to plastic in DMEM containing 5% FBS. The nonadherent fraction was washed and the adherent Kupffer cells were collected for further experiments.

### Isolation and culture of primary mouse hepatocytes

Mouse hepatocytes were isolated using a collagenase perfusion method as previously reported 71. In brief, livers from wild-type mice were perfused with Hanks' balanced salt solution (HBSS) buffer through the portal vein and digested with collagenase IV. After washing with HBSS, hepatocytes were seeded into collagen type I-coated plates and cultured in DMEM supplemented with 10% FBS and 1% penicillin/streptomycin. 3 h later, the medium was replaced with serum-free DMEM. On the next day, cells were collected for qPCR assays.

### Isolation of hepatic stellate cells

Hepatic stellate cells (HSCs) were isolated from wild-type mice as previously described 72. In brief, mouse liver was perfused with pronase E and 0.1% collagenase IV (dissolved in phosphate buffer saline) through the inferior vena cava. The liver was then digested with 0.2% collagenase IV, and filtered through a 70-µm mesh. Cells were separated using a Nycodenz gradient centrifugation. HSCs were collected, seeded into polylysine-coated plates, and cultured in DMEM containing 10% FBS and 1% penicillin/streptomycin.

### Synchronized cells

Synchronization (serum shock) experiments with cultured cells were performed as previously described 14. BMDMs were cultured in RPMI medium containing 10% FBS and 1% penicillin-streptomycin. On the next day, culture medium was replaced with serum-free medium. 12 h later, 50% horse serum was added for 2 h and the medium was changed back to serum-free medium. Cells were harvested for RNA extraction at specific time points (0, 4, 8, 12, 16, 20 and 24 h). Notably, BMDMs were primed with LPS (500 ng/mL) for 3 h before cell harvest.

### *ASC* oligomerization assay

Transfected and control BMDMs were lysed in AO buffer (containing 150 mM KCl, 20 mM HEPES-KOH, 1% Triton-X 100 and 1% protease inhibitor). The lysate was centrifugated at 5000 g for 15 min. The pellets (Triton-insoluble fraction) were collected in PBS, and cross-linked with 2 mM disuccinimidyl suberate for 30 min. After centrifugation (5000 g for 15 min), the pellets were subjected to Western blotting analysis.

### FISH (fluorescence *in situ* hybridization)

Subcellular distribution of *Platr4* was assessed using a FISH kit according to the manufacturer's instructions (GenePharma, Shanghai, China). In brief, BMDMs were fixed in 4% paraformaldehyde, and hybridized with 10 nM *Platr4* probe in a hybridization buffer. Cells were washed with saline sodium citrate and counterstained with DAPI. Images were obtained using a laser scanning microscope (Carl Zeiss, Oberkochen, Germany). The forward and reverse sequences of FISH probe were 5'-CTGTGACTTCTCCAGGGCAG and 5'-GGACAATCTCACGTGCTCCA, respectively.

### RIP (RNA immunoprecipitation)

RIP assay was performed using a Magna RIP kit (Millipore, Bedford, MA). In brief, livers were homogenized and lysed in lysis buffer containing an RNase inhibitor and a protease inhibitor cocktail. After centrifugation (14000 rpm for 10 min), the supernatant was incubated with magnetic beads bound with anti-Rxrα or normal rabbit IgG antibody in immunoprecipitation buffer overnight at 4 °C. Beads were then washed with RIP washing buffer containing proteinase K. The immunoprecipitates were subjected to RNA extraction and qPCR assays.

### RNA pull-down

Nuclear proteins were prepared from mouse liver using a nuclear protein extraction kit according to the manufacturer's protocol (Beyotime, Shanghai, China). Full-length *Platr4* and five truncated versions of *Platr4* were cloned into T7 promoter-based vector and transcribed using TranscriptAid T7 High Yield Transcription kit (Thermo Scientific, Madison, WI), followed by RNA purification. Purified RNA was biotinylated using a Pierce Magnetic RNA-Protein Pull-down kit (Thermo Scientific, Madison, WI). Biotinylated RNA was incubated with streptavidin magnetic beads at room temperature. After 30 min, beads were incubated with 5 mg nuclear proteins at 4 °C for 1 h. RNA binding proteins were eluted from the beads with elution buffer. An aliquot of pull-down products were subjected to mass spectrometric analysis, while the remaining were analyzed by Western blotting.

### Immunofluorescence analysis

Immunofluorescence analysis was performed as described in our previous publication 54. Cells were fixed in 4% paraformaldehyde, permeated in 0.1% Triton X-100, and blocked with 5% BSA. Then, cells were sequentially incubated with primary antibody and Alexa Fluor 488-conjugated or Alexa Fluor 594-conjugated anti-rabbit secondary antibody, followed by DAPI staining. Images were obtained using a laser scanning microscope (Carl Zeiss, Oberkochen, Germany).

### RNA-seq (RNA-sequencing)

Three normal mice and three steatohepatitis mice were sacrificed at ZT2, and the livers were isolated. Total RNA was extracted using RNAiso Plus reagent. Two RNA pools (i.e., experimental samples) for normal and steatohepatitis groups were assembled by mixing equal amounts of RNA from three animals. The RNA pools were subjected to library construction and RNA-seq as described in our previous publication 54. In the second set of experiments, livers were collected from three wild-type and three *Rev-erbα^-/-^* mice at each of circadian time points (ZT6 and ZT10). RNA samples were pooled from three animals, and subjected to RNA-seq analysis. Additionally, RNA-seq was performed with BMDM samples (*Platr4*-transfected and LPS-stimulated BMDMs versus control BMDMs). Transcriptomics data analysis was performed as described 54.

### qPCR, Western blotting, luciferase reporter assay, EMSA, ChIP and Co-IP

Standard methods were used for qPCR, Western blotting, luciferase reporter assay, EMSA, ChIP and Co-IP, and experimental procedures have been described in our previous publications 54,65,73. The sequences for oligonucleotides (primers) used in qPCR and ChIP are provided in Tables S3-4. The amplified sequences in qPCR analysis of *Platr4* are specifically located within the *Platr4*-203 (an amplified product from 1211 to 1288 bp in this isoform,* Platr4* primers shown in Table S3). Copy number quantification was performed using *in vitro*-synthesized *Platr4* for the creation of a standard curve.

### Statistical analysis

Data are presented as mean ± SD. Statistical significance was determined using Student's t test or ANOVA (one-way or two-way with Bonferroni post hoc test). The level of significance was set at *p <*0.05 (*).

## Supplementary Material

Supplementary figures and tables.

## Abbreviations

AAV8: adeno-associated virus serotype 8
ALT: alanine aminotransferase
Asc: apoptosis-associated speck-like protein containing a CARD
α-SMA: α-smooth muscle actin
ASO: antisense oligonucleotide
AST: aspartate transaminase
ATP: adenosine triphosphate
BMAL1: brain and muscle ARNT-like 1
BMDMs: bone marrow derived macrophages
Col1a1: collagen, type I, alpha 1
ChIP: chromatin immunoprecipitation
CLOCK: circadian locomotor output cycles kaput
Co-IP: co-immunoprecipitation
CRY: cryptochrome
DAPI: 4',6-diamidino-2-phenylindole
Dbp: D-box binding protein
E4bp4: E4 promoter-binding protein 4
EMSA: electrophoretic mobility shift assay
FISH: fluorescence *in situ* hybridization
HSCs: hepatic stellate cells
LncRNA: long non-coding RNAs
LPS: lipopolysaccharide
MCD: methionine-choline-deficient
MCP-1: monocyte chemoattractant protein-1
MPO: myeloperoxidase
NAFLD: nonalcoholic fatty liver disease
NASH: nonalcoholic steatohepatitis
NAS: NAFLD activity score
NF-κB: nuclear factor kappa-B
*NLRP3*: NOD- LRR- and pyrin domain-containing protein 3
IL: interleukin
PER: period
qPCR: quantitative polymerase chain reaction
RevRE: REV-ERB response element
RIP: RNA immunoprecipitation
RNA-seq: RNA sequencing
Rxrα: retinoid X receptor alpha
TBG: liver-specific thyroxine-binding globulin
Tgf-β1: transforming growth factor beta 1
Tnf: tumor necrosis factor
ZT: zeitgeber time
