# Supplementary material for: Oscillating lncRNA Platr4 regulates NLRP3 inflammasome to ameliorate nonalcoholic steatohepatitis in mice
Source: Theranostics. 2021 Jan 1;11(1):426–44. doi: 10.7150/thno.50281 (PMC7681083; doi:10.7150/thno.50281)
Supplement: Supplementary file 1 — Supplementary figures and tables. [file thnov11p0426s1.pdf]

Supplementary Information

Oscillating lncRNA *Platr4* regulates NLRP3 inflammasome to ameliorate nonalcoholic steatohepatitis in mice

Figure S1. Plasma ALT/AST activities and hepatic triglyceride level in wild-type (WT) mice fed a normal or MCD diet. Data are mean ± SD (*n* = 8). *p < 0.05 (t-test).

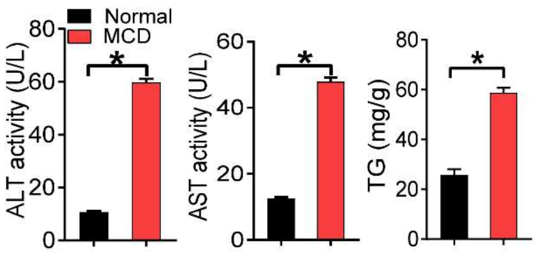

Figure S2. Hepatic mRNA expressions of inflammatory factors (*IL-1*β, *IL-6, Tnf*α and *IL-18*) (left panel) and pro-fibrotic genes (α*-SMA, Col1a1* and *Tgf-*β*1*) (right panel) in WT mice fed a normal or MCD diet. Data are mean ± SD (*n* = 8). *p < 0.05 (t-test).

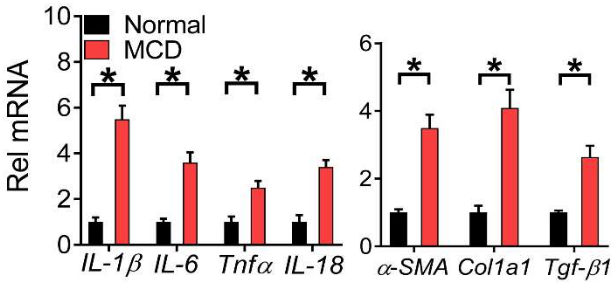

Figure S3. KEGG analysis of MCD-induced differentially expressed genes in the livers of WT mice.

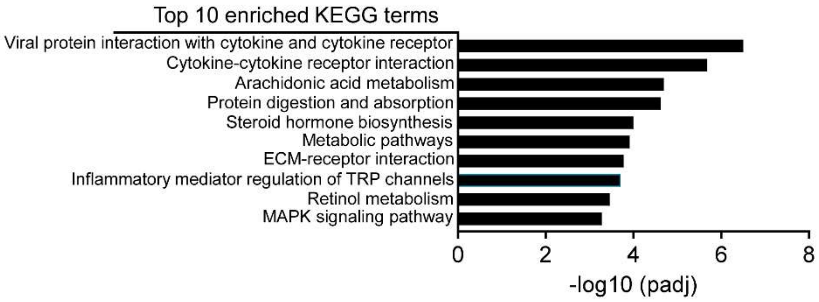

Figure S4. (A) Predicted coding probability of *Platr4*, *Neat1* and *Gapdh* using the CPAT tool. **(B)** Coding potential scores of *Platr4*, *Neat1* and *Gapdh* predicted by the CPC calculator. CPAT, coding potential assessment tool; CPC, coding potential calculator.

A

CPAT

LncRNA/gene	C/NC	Coding probability
Platr4	noncoding	0.02
Neat1	noncoding	0.07
Gapdh	coding	0.10

B

CPC

LncRNA/gene	C/NC	Coding potential score
Platr4	noncoding	-0.20
Neat1	noncoding	-0.06
Gapdh	coding	13.62

Figure S5. GO analysis of differentially expressed genes in *Platr4*-overexpressed versus control BMDMs after 8-h LPS (500 ng/ml) treatment.

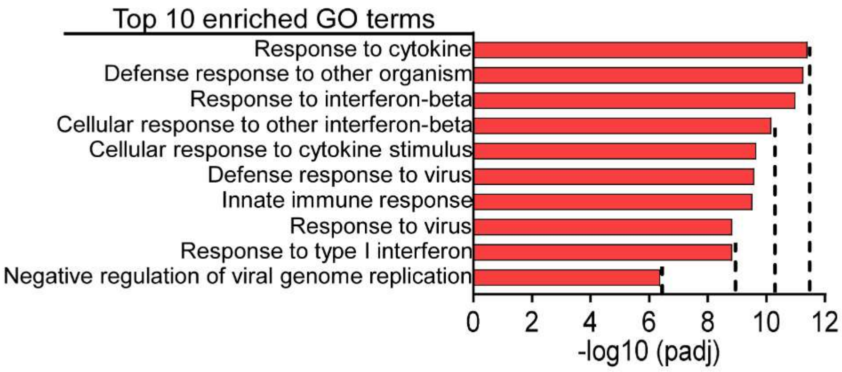

Figure S6. (A) Schematic of CRISPR/Cas9-mediated genome engineering for *Platr4* knockout mice. **(B)** Validation of *Platr4* knockout efficiency. Data are mean ± SD ($n = 8$). N.D., not detected.

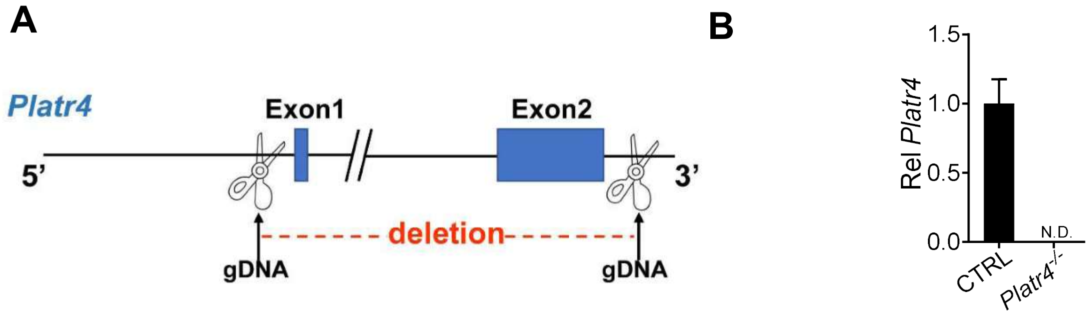

Figure S7. Effects of *Platr4* knockout on expression of the neighbor gene *Jade1*. Data are mean ± SD (*n* = 8).

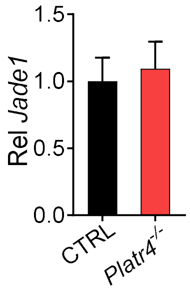

Figure S8. Effects of Rxrα knock-down on NF-κB target genes (*Nlrp3, IL-1β, IL-6, Tnfα* and *IL-18*) in LPS-stimulated BMDMs. Data are mean ± SD (*n* = 3). *p < 0.05 (t-test).

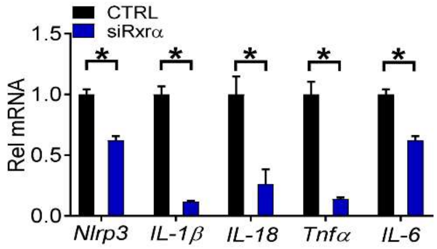

Figure S9. (A) Effects of Rxrα on the NF-κB-dependent reporter activity in BMDMs. Data are mean ± SD (*n* = 6). *p < 0.05 (one-way ANOVA with Bonferroni post hoc test). **(B)** Effects of Rxrα and/or p65 on the *Nlrp3* reporter activity in BMDMs. Data are mean ± SD (*n* = 6). *p < 0.05 (one-way ANOVA with Bonferroni post hoc test).

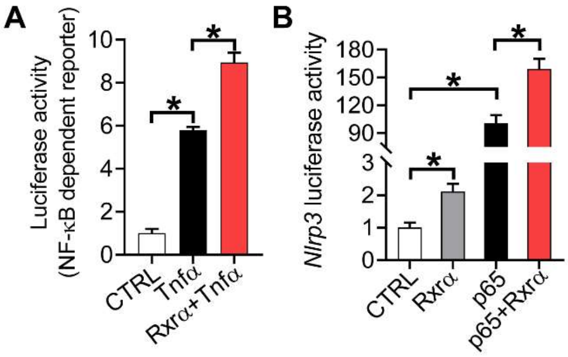

Figure S10. Recruitment of p65 protein to the κB sites of *Nlrp3* and *mCSF* promoter (two NF-κB target genes) in Rxrα knocked-down BMDMs. Data are mean ± SD (n = 3). *$p < 0.05$ (two-way ANOVA and Bonferroni post hoc test).

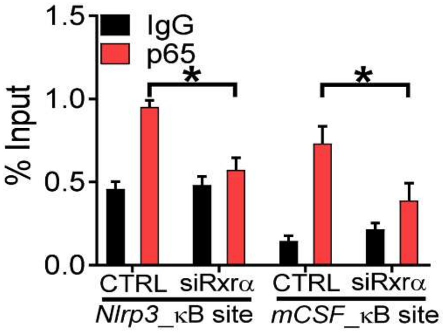

Figure S11. Effects of *Platr4* overexpression and knockdown on *Rxrα* expression in BMDMs. Data are mean ± SD (*n* = 3).

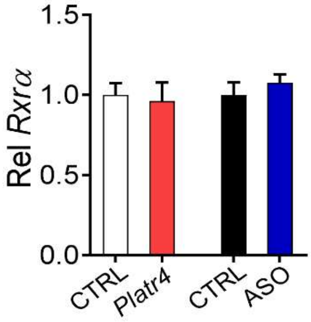

Figure S12. Hepatic *Platr4* expression in wild-type mice on day 15 after a single injection of AAV8.TBG.*Platr4* or control vector. Data are mean ± SD (n = 8). *p < 0.05 (t-test).

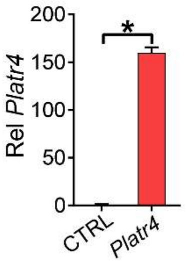

Table S1. List of candidate proteins interacting with *Platr4* from mass spectrometric analysis

Candidate proteins	Mass	Score	emPAI
Mthfd1	82503	189	0.26
Fdps	13980	88	0.24
Psmd7	36574	76	0.09
Taok3	64413	75	0.10
Sntb1	58444	57	0.06
Hsd17b2	42436	56	0.16
**Rxrα**	**51925**	**50**	**0.13**
Eif1b	11465	48	0.30
Ube2t	8184	42	0.43
Metap2	21275	38	0.34
Bard1	85683	34	0.04
Ilf3	18830	33	0.18
Psmd11	9907	33	0.35
Pym1	22676	32	0.15
Eif3e	52587	30	0.06
Rbm42	47298	27	0.07
Pxmp4	10473	22	0.33

Table S2. Sequences of antisense oligonucleotide (ASO) and siRNA

Name	Sequences (5'-3')
Negative control (ASO-NC)	TGGGACGCCTGGGTACAC
ASO	CAAGTAAGATGGCTCAGTGC
Negative control (si-NC)	UUCUCCGAACGUGUCACGUTT
siRev-erbα	CUUCGUUGUUCAACGUGAATT
siBmal1	GCUCUUUCUUCUGUAGAAUTT
siRxrα	CCAUCGACACCUUCCUCAUdTdT

Table S3 Sequences of primers for qPCR and PCR analyses

Name	Forward (5'-3' sequence)	Reverse (5'-3' sequence)
Platr4	ATGGAGGAGGAAGTGAAA	TACCCAAGAAGCCTGTTA
Neat1	TTGGGACAGTGGACGTGTGG	TCAAGTGCCAGCAGACAGCA
β-actin	TTGAACATGGCATTGTTACCAA	TGGCATAGAGGTCTTTACGGA
Hmbs	CCGAGCCAAGGACCAGGATA	CTCCTTCCAGGTGCCTCAGA
Bmal1	CTCCAGGAGGCAAGAAGATTC	ATAGTCCAGTGGAAGGAATG
Rev-erbα	TTTTTCGCCGGAGCATCCAA	ATCTCGGCAAGCATCCGTTG
IL-1β	AATGCCACCTTTTGACAGTGATG	AGCTTCTCCACAGCCACAAT
Nlrp3	ATTACCCGCCCGAGAAAGG	TCGCAGCAAAGATCCACACAG
Asc	GACAGTACCAGGCAGTTCGT	GAGTCCTTGCAGGTCAGGTT
IL-6	ATCCAGTTGCCTTCTTGGGACTGA	TAAGCCTCCGACTTGTGAAGTGGT
Tnfα	AGGGTCTGGGCCATAGAACT	CCACCACGCTCTTCTGTCTAC
IL-18	TCAAAGTGCCAGTGAACCCC	GGTCACAGCCAGTCCTCTTAC
Rxrα	CTGTGCTATCTGTGGGGACC	AGGCAGTCCTTGTTGTCTCG
p65	ACTGCCGGGATGGCTACTAT	TCTGGATTCGCTGGCTAATGG
α-SMA	TGTGCTGGACTCTGGAGATG	GAAGGAATAGCCACGCTCAG
Col1a1	GCTCCTCTTAGGGGCCACT	CCACGTCTCACCATTGGGG
Tgf-β1	CTCCCGTGGCTTCTAGTGC	GCCTTAGTTTGGACAGGATCTG
Jade1	GCGTTAGGCTACGTCGATATCC	ACGCAGCATCCATATCATTGAG
**For PCR genotyping**		
Platr4	TCAAGAGCGGACTGCACCGAAG	TCTGGCCTCTTCTCCTTGGACATC
Platr4-He/Wt	TGCATTTGGTTTCTGTGTTCTTTAATCTC	

Table S4. Sequences of primers for ChIP assays

Name	Forward (5'-3' sequence)	Reverse (5'-3' sequence)
*Asc*_κB site	CAGCCTAGCCAAAAAGCCAA	GCCCGCCAAGACCAGG
*Asc*_NB	CTCCTGCCTCTGTCTCTCGTA	CCAATACCCAGCATAGGGCTT
*Nlrp3*_κB site	GAGCCCTGAGGTTTCACTTTTTC	AGCTTGCATCACTGTCAAAGACG
*Bmal1*_REV	GATTGGTCGGAAAGTAGGTTAGT	GTCGCACCACTAACCTACTTTCC
*Platr4*_REV1	ACTCACAACTTCCACTACTTCGT	CTTTGAGCCCATGAAAATACACA
*Platr4*_REV2	GAGCGGACTGCACCGAAG	GGAAGGGGATTCTGGGTAGTG
*Platr4*_NB	ACATAGTCTTTAGTCTTTTGAGGC	AGCAGTGGAGTCTGAAGGAT
*Platr4*_κB site1	TTCCCTTTCCTCATTTCC	GGAGTAGGTGGGTTGGTG
*Platr4*_κB site2	GTGTAGTCCCGGAAATCC	CCTTGCTAGACGGTGGTG
*mCSF*_κB site	GGGCCTCTGGGGTGTAGTAT	CCGAGGCAAACTTTCACTTT
